# A multidimensional atlas of human glioblastoma-like organoids reveals highly coordinated molecular networks and effective drugs

**DOI:** 10.1038/s41698-024-00500-5

**Published:** 2024-01-26

**Authors:** Changwen Wang, Meng Sun, Chunxuan Shao, Lisa Schlicker, Yue Zhuo, Yassin Harim, Tianping Peng, Weili Tian, Nadja Stöffler, Martin Schneider, Dominic Helm, Youjun Chu, Beibei Fu, Xiaoliang Jin, Jan-Philipp Mallm, Moritz Mall, Yonghe Wu, Almut Schulze, Hai-Kun Liu

**Affiliations:** 1 Division of Molecular Neurogenetics, German Cancer Research Center (DKFZ); The DKFZ-ZMBH alliance, Im Neuenheimer Feld 581, 69120 Heidelberg, Germany; 2 Faculty of Medicine, Heidelberg University, Im Neuenheimer Feld 672, 69120 Heidelberg, Germany; 3 Department of Thyroid Surgery, The First Affiliated Hospital, School of Medicine, Zhejiang University, 310003 Hangzhou, China; 4 Shanghai Institute for Advanced Immunochemical Studies, ShanghaiTech University, 201210 Shanghai, China; 5 School of Life Science and Technology, ShanghaiTech University, 201210 Shanghai, China; 6 Division of Tumor Metabolism and Microenvironment, German Cancer Research Center (DKFZ), Im Neuenheimer Feld 581, 69120 Heidelberg, Germany; 7 Proteomics Core Facility, German Cancer Research Center (DKFZ), Im Neuenheimer Feld 580, 69120 Heidelberg, Germany; 8 Faculty of Biosciences, Heidelberg University, Im Neuenheimer Feld 234, 69120 Heidelberg, Germany; 9 Department of Ophthalmology, Ninth People’s Hospital, Shanghai Jiao Tong University School of Medicine, 200025 Shanghai, China; 10 Shanghai Key Laboratory of Orbital Diseases and Ocular Oncology, 200025 Shanghai, China; 11 Single-cell Open Lab, German Cancer Research Center (DKFZ), Im Neuenheimer Feld 280, 69120 Heidelberg, Germany; 12 Cell Fate Engineering and Disease Modeling Group, German Cancer Research Center (DKFZ) and DKFZ-ZMBH Alliance, 69120 Heidelberg, Germany; 13 HITBR Hector Institute for Translational Brain Research gGmbH, 69120 Heidelberg, Germany; 14 Central Institute of Mental Health, Medical Faculty Mannheim, Heidelberg University, 68159 Mannheim, Germany

**Keywords:** CNS cancer, Cancer genetics, Tumour heterogeneity, Drug screening

## Abstract

Recent advances in the genomics of glioblastoma (GBM) led to the introduction of molecular neuropathology but failed to translate into treatment improvement. This is largely attributed to the genetic and phenotypic heterogeneity of GBM, which are considered the major obstacle to GBM therapy. Here, we use advanced human GBM-like organoid (LEGO: Laboratory Engineered Glioblastoma-like Organoid) models and provide an unprecedented comprehensive characterization of LEGO models using single-cell transcriptome, DNA methylome, metabolome, lipidome, proteome, and phospho-proteome analysis. We discovered that genetic heterogeneity dictates functional heterogeneity across molecular layers and demonstrates that *NF1* mutation drives mesenchymal signature. Most importantly, we found that glycerol lipid reprogramming is a hallmark of GBM, and several targets and drugs were discovered along this line. We also provide a genotype-based drug reference map using LEGO-based drug screen. This study provides new human GBM models and a research path toward effective GBM therapy.

## Introduction

Oncogenic genetic alteration is a fundamental hallmark of human cancers and has been utilized to characterize genotype-specific molecular features, which form the basis for personalized treatment of cancer patients^1,2^. Based on these efforts, genotype-based personalized cancer treatment options are already available for many human cancers, i.e., breast cancer, lung cancer, and leukemia^1^. However, it remains challenging to expand personalized treatment to most cancer patients^3^.

GBM is the most malignant type of primary brain cancer and was one of the first tumor entities selected for The Cancer Genome Atlas (TCGA) project^4,5^. With the continuous efforts in genomic analysis of GBM, it has been suggested that GBM is a heterogeneous group of diseases of different molecular subtypes based on RNA expression, DNA methylation, or recently via multi-omics analysis^4,6–8^. Single-cell RNA-sequencing (scRNA-seq) analysis of human GBM identified intratumoral heterogeneity of GBM, which provides a single-cell molecular description of human GBM; it was suggested that the GBM cells are of high plasticity, which may switch among the molecular phenotypes^9,10^. However, it must be noted that how tumor genotype contributes to the molecular phenotype-related plasticity remains unclear, i.e., *NF1* mutation in human GBM is associated with a mesenchymal feature, but this has not been verified in animal models^4,5^. And it is much more challenging to perform in-depth single-cell DNA sequencing. In contrast to the rapid development of molecular characterization of GBM, the clinical treatment options for human GBM patients remain to be neurosurgery, plus radiotherapy and temozolomide(TMZ)-based chemotherapy^11^. There is a clear gap between the comprehensive molecular description of GBM and treatment improvement, which needs to be highly prioritized for future GBM research.

A genome-based personalized treatment of cancer patients requires a solid understanding of genotype-specific cancer pathway dependency and actionable target identification. Model systems of GBM have been utilized to systematically analyze and compare differences in cancer cells with different mutation combinations. Genetically modified mouse models have been used to determine the function of selected genes and identify the cell of origin in brain tumors^12^. However, mouse models often do not represent the molecular pathology of human tumors^13^. Patient-derived xenograft (PDX) models or organoids harbor patient tumor cells. Still, they are limited by complex genetic background variations, differences in treatment histories, and, most importantly, the lack of suitable controls^14^. Most importantly, the tumor growth characteristics identified in PDX models were found to be more dependent on the mouse strain than tumor type^15^, suggesting the PDX model may generate many artificial readouts irrelevant to primary human tumors.

The recent development of organoid technology coupled with gene editing by CRISPR/Cas9 allows the rapid generation of genetic mutations in human-derived tissues to model cancer progression^16^. Initial attempts were made using induced pluripotent stem cells (iPSCs)-derived cerebral organoids to generate glioma-like organoids^17,18^. This model provides the opportunity to develop genetically customized GBM models derived from single iPSC clones. Therefore, a rigorous follow-up analysis can be performed using this experimental system.

Here we generated a set of iPSC-based human GBM-like organoid models (**LEGO**: Laboratory Engineered Glioblastoma-like Organoid) based on CRISPR/Cas9 engineered loss of tumor suppressors, which are frequently mutated in human GBM patients. Comprehensive analysis of LEGOs demonstrates their great potential in identifying new molecular features in cancer cells, providing a path toward personalized treatment of human GBM.

## Results

### Generation of LEGOs with defined genetic mutations

**Fig. 1:**
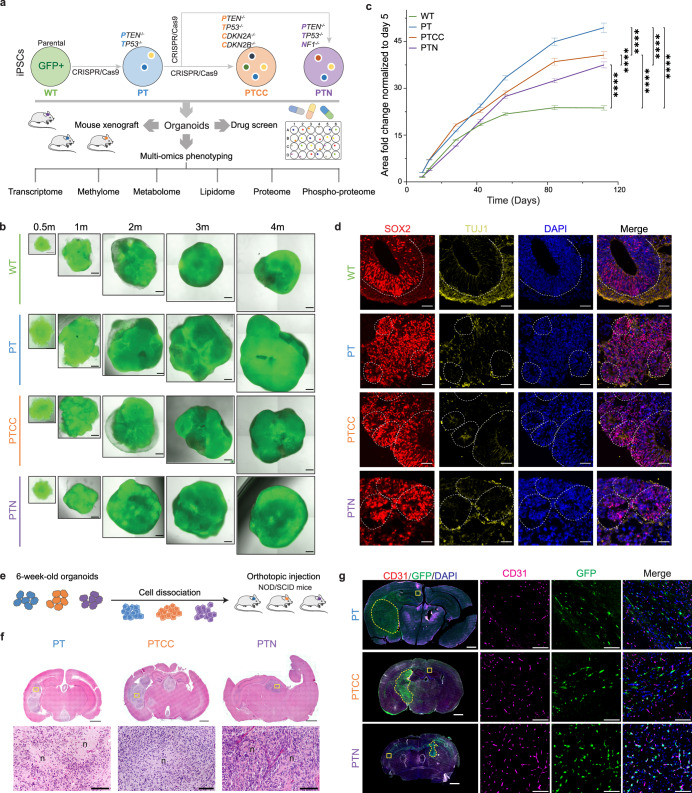
Generation and histological characterization of LEGOs with defined genetic mutations. **a** Schematic illustration of experimental procedures in this study. **b** Representative images showing the morphology of organoids at different ages. **c** Organoid growth curves normalized to respective average size on day 5. *P* values of the comparison between different groups of organoids were calculated by Two-way ANOVA. Data are represented as mean ± SEM for quantifying the 2D area of at least 65 organoids from at least four independent batches at each time point. **d** Representative immunofluorescent staining images of 1-month-old organoids stained with SOX2 and TUJ1. White circles show rosette-like structures. Scale bars, 50 μm. **e** Schematic diagram illustrating mouse xenograft workflow. **f** Representative H&E staining images of brain tumors in mouse xenografts. “n” in the enlarged image marks necrotic areas. Scale bar, 1000 μm for the overview, and 100 μm for insets (**g**). Representative immunofluorescent images of the tumor-infiltrating area stained with GFP and CD31. Note a strong association between GFP (green) and CD31(magenta) -positive cells in the PTN xenografts. Scale bars, 1000 μm for overview images, and 100 μm for insets. See also Supplementary Fig. 1 and Supplementary Table 1.

We used human iPSC-derived organoids to dissect the functional consequences of genetic heterogeneity in GBM (Fig. 1a). Using CRISPR/Cas9, we generated a spectrum of mutation combinations (**PT**: *PTEN*^*-/-*^; *TP53*^*-/-*^, **PTCC**: *PTEN*^*-/-*^; *TP53*^*-/-*^; *CDKN2A*^*-/-*^; *CDKN2B*^*-/-*^, **PTN**: *PTEN*^*-/-*^; *TP53*^*-/-*^; *NF1*^*-/-*^), which are among the most frequently mutated tumor suppressors in GBM patients^5^, in two iPSCs derived from healthy donor, one cell line expressing GFP. The knockout of individual genes was confirmed by Western blotting and sequencing (Supplementary Fig. 1a, b). All iPSCs clones grew well except that the PTN clone showed signs of differentiation, which was reported previously and could be controlled by MEK inhibitor PD0325901^19^. We conducted staining of all iPSCs using pluripotent stem cell markers such as SOX2 (SRY-box transcription factor 2), Nanog (nanog homeobox), and OCT3/4 (octamer-binding transcription factor 3/4). The results from staining confirmed that the pluripotent nature of these iPSCs were not changed by the knockout of tumor suppressors (Supplementary Fig. 1c). These iPSCs were then differentiated into organoids with a previously described cerebral organoid protocol^20^. Although starting from the same number of cells, all the LEGOs grew faster and more extensively than WT organoids (Fig. 1b, c), indicating the activation of cell proliferation and growth pathways following the oncogenic mutations. Interestingly, the size of PT organoids was the biggest among the three mutant groups (Fig. 1c).

The histological analysis showed that the LEGOs exhibited similar structures compared to the WT organoids, indicated by the expression of SOX2 and TUJ1 (neuron-specific class III beta-tubulin) (Fig. 1d). However, all mutant LEGOs show an increased stem/progenitor population (Fig. 1d). We also performed the staining using FABP7 (fatty acid binding protein 7), DCX (doublecortin) and SOX9 (SRY-box transcription factor 9) (Supplementary Fig. 1d). The histological analysis showed that the LEGOs exhibited more expression of FABP7 and SOX9, less expression of DCX than WT organoids. H&E staining revealed nuclear atypia in LEGOs after more than 1 month of culture (Supplementary Fig. 1e), indicating signs of malignant transformation. To investigate whether the LEGOs are tumorigenic in vivo, we performed xenograft experiments, as illustrated in Fig. 1e. All LEGOs initiated fatal brain tumors upon xenograft, while WT xenografts survived significantly longer than LEGO xenografts without forming lethal tumors (Fig. 1f, Supplementary Fig. 1f, and Supplementary Table 1). Interestingly, the H&E staining showed survival of grafted cells (Supplementary Fig. 1g), same as what has been reported before^20,21^. On the other hand, the grafted GFP+ LEGO cells showed infiltrative and angiogenic growth patterns (Fig. 1g and Supplementary Fig. 1h). Moreover, PTN xenografts exhibited a more infiltrative growth pattern with tumor cells migrating to the other hemisphere and being tightly associated with blood vessels (Fig. 1g), suggesting that loss of *NF1* results in a more invasive phenotype, which is a feature of the mesenchymal molecular phenotype of human GBM. In addition, all grafts expressed markers, like astrocyte marker GFAP (glial fibrillary acid protein), neural stem cell marker Nestin, and cell proliferation marker Ki67 (Supplementary Fig. 1i), the tumors also show signs of necrosis, which is a hallmark for GBM (Fig. 1f). These results demonstrate that the LEGOs are GBM-like organoids and are tumorigenic in vivo.

### ScRNA-seq analysis reveals shared and genotype-specific alterations during early tumor development

One of the advantages of cerebral organoids is that they contain heterogeneous neural cell populations and maintain differentiation hierarchies^20^, thus can be used to study cellular heterogeneity and plasticity. To fully characterize the LEGOs on the single-cell level and to understand how different genetic mutations affect cellular heterogeneity, we performed scRNA-seq on 1- and 4-month-old LEGOs. In total, we obtained results from 70617 cells for further analysis.

**Fig. 2:**
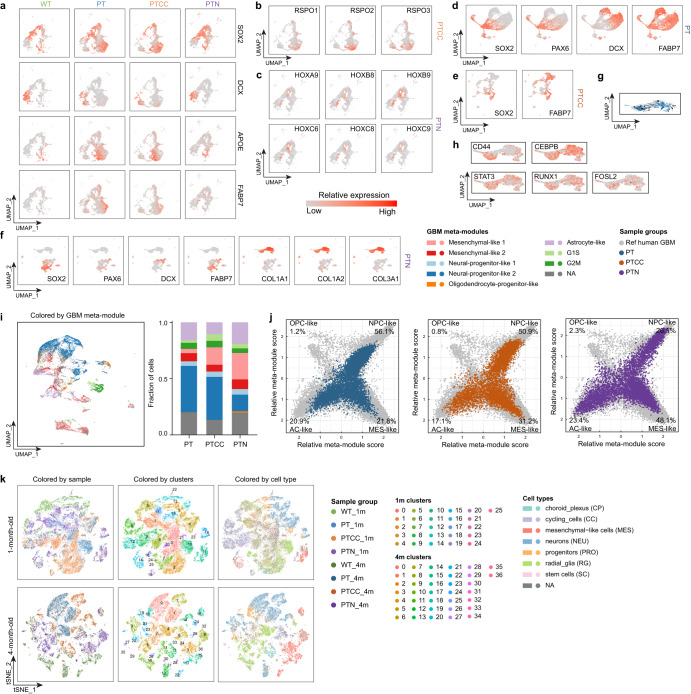
ScRNA-seq analysis reveals shared and genotype-specific alterations during early GBM development. **a** UMAP plots show the relative expression of lineage markers in 1-month-old organoids. **b** UMAP plots for RSPO genes in 1-month-old PTCC organoids. **c** UMAP plots for HOX genes in 1-month-old PTN organoids. UMAP plots for lineage markers in 4-month-old PT (**d**), PTCC (**e**), and PTN (**f**) organoids. **g** RNA velocity analysis of the mesenchymal-like clusters in PTN organoids. **h** UMAP plots of the mesenchymal-related marker genes in PTN organoids. **i** UMAP representation of LEGO models, color-coded by GBM meta-modules, with cell proportion displayed on the right. **j** Mapping of relative meta-module scores of LEGOs to human GBM meta-module scores. **k** t-SNE plots of the 1- and 4-month-old organoids colored by sample groups, clusters or cell types. See also Supplementary Fig. 2.

We next performed UMAP (uniform manifold approximation and projection) analysis to visualize cell differentiation trajectory^21^. UMAP of 1-month-old LEGOs show two major lineages (neuron and astrocyte), which was confirmed by the expression of immature neuronal marker DCX, astrocytic marker FABP7 and APOE (apolipoprotein E), and neural stem/progenitor marker SOX2 (Fig. 2a and Supplementary Fig. 2a). The 1-month-old WT organoids mainly differentiated toward the neuronal lineage, whereas the PT and PTCC organoids switched to astrocytic differentiation (Fig. 2a and Supplementary Fig. 2a). The PTN organoids exhibited limited neuronal differentiation and reduced astrocytic differentiation (Fig. 2a and Supplementary Fig. 2a), suggesting a general blockage of neural differentiation. We also observed increased expression of neural stem/progenitor markers like SOX2 in all the LEGOs, indicating differentiation blockage upon loss of tumor suppressors, consistent with staining (Fig. 1d and Supplementary Fig. 2a). Interestingly, PTCC organoids highly express WNT regulators in the glial progenitor population, suggesting the activation of the WNT pathway upon loss of CDKN2A/2B (Fig. 2b). Surprisingly, the PTN organoids activate several HOX transcription factors in the stem cell clusters (Fig. 2c). The HOX genes have been reported to be involved in the induction of EMT (epithelial-mesenchymal transition) in other cancers^22^, and they are not expressed in normal neural cells (Supplementary Fig. 2b), which highly suggests that PTN organoids may activate a non-neural transcriptional program to acquire a more aggressive phenotype.

The scRNA-seq results from 4-month-old organoids demonstrate that the PT organoids are dominated by two major cell populations, one shows high expression of stem/progenitor cell markers like SOX2 and PAX6 (paired box 6), and the other express the immature neuron marker DCX and the astrocyte marker FABP7 (Fig. 2d), indicating a proneural-like tumor cell feature. PTCC organoids also maintain a differentiation trajectory towards the FABP7 astrocytic lineage from the SOX2-positive stem cell cluster (Fig. 2e). Strikingly, there are two major differentiation lineages in PTN organoids; one is the neural lineage, as indicated by the expression of SOX2, PAX6, and DCX (Fig. 2f), while the other lineage highly expresses collagen genes and can be divided into two clusters (Fig. 2f). The RNA velocity analysis suggested a possible differentiation hierarchy between the two clusters (Fig. 2g), with the stem-cell-like cluster expressing CD44 (Fig. 2h). Moreover, this lineage was positive for mesenchymal master regulators like STAT3 (signal transducer and activator of transcription 3), C/EBPB (CCAAT enhancer binding protein beta), RUNX1 (RUNX family transcription factor 1), and FOSL2 (FOS like 2, AP-1 transcription factor subunit) (Fig. 2h)^23^. We also found that these cells express unique markers like PAX7 (paired box 7) and CHODL (chondrolectin) (Supplementary Fig. 2c), which can potentially be used to identify these cells in human cancers.

Next, we conducted further analysis of the single-cell RNA sequencing data to enable a more comprehensive comparison of LEGOs and human GBM. To achieve this, we employed two distinct gene signature sets: the GBM cell state and GBM meta-modules, both derived from single-cell RNA sequencing data of human GBM patients^9,24,25^. The LEGOs contain major tumor cell populations such as “stem-like”, “proliferating stem-like”, and “differentiated-like” cells (Supplementary Fig. 2d). Moreover, PT was dominated by the “stem-like” cell population resembling the proneural subtype (Supplementary Fig. 2e)^24^. PTN showed an increased proportion of “differentiated-like” cells mimicking the mesenchymal subtype (Supplementary Fig. 2e)^24^. Moreover, we calculated the single cell meta module score^9^ of the mutant organoids and found that PT and PTCC organoids were dominated by the neural progenitor-like cells and PTN organoids were dominated by the mesenchymal-like cells (Fig. 2i). However, we were unable to locate specific human GBM single-cell RNA sequencing data that precisely matched the mutations established in our model, underscoring the complex mutational background of patient GBM data and the challenge of quantitatively assessing the phenotypic impact of individual genes and highlighting the advantage of LEGO models. Furthermore, we conducted a comparative analysis by aligning the relative meta-module scores^9^ of our LEGO models with the corresponding scores^9^ in human GBM (Fig. 2j). This comparison revealed a substantial overlap, reinforcing the strong resemblance between our LEGO models and human GBM, and *NF1* mutation drives a mesenchymal-like lineage during organoid development, and it will be interesting to trace the origin of these cells in the future.

The LEGO models recapitulated critical features of cellular heterogeneity discovered in human GBM. The advantage that all LEGOs were derived from the exact iPSC clone with defined mutations allows us to further analyze how genetic heterogeneity contributes to cellular heterogeneity, which was not possible based on previous models. We first used t-SNE (t-distributed stochastic neighbor embedding) analysis for cell cluster analysis of all LEGOs together. To distinct different cell types in our organoids, we enriched our organoid cell type data by incorporating cluster signatures derived from cerebral organoids that were cultivated using the same culturing method^26^. Our analysis unveiled the presence of distinct cell types within our organoids, which include neurons, radial glia cells, and stem cells (Fig. 2k). It is evident that these different cell types are well-separated, particularly as the organoids reach 4 months of age (Fig. 2k). What’s particularly intriguing is that although cells from different genotypes exhibit the same cell type signatures, for instance, mesenchymal-like cells (cluster 3, 8, 15, 16, 17), these cells from different genotypes cluster separately (cluster 3, 8 from PTN, cluster 15, 17 from WT) (Fig. 2k and Supplementary Fig. 2f). This phenomenon is also observable in other cell types such as neurons and radial glia cells (Fig. 2k and Supplementary Fig. 2f).

In conclusion, using Single-cell RNA sequencing (ScRNA-seq), we observed that LEGO models accurately replicate essential aspects of cellular diversity found in human GBM, and that genetic mutations strongly influence cell phenotypes. Nevertheless, the stem cell differentiation hierarchy driven by neurodevelopmental programs persists throughout tumor formation.

### DNA methylome analysis reveals genotype-dependent progressive changes of DNA methylation during gliomagenesis

**Fig. 3:**
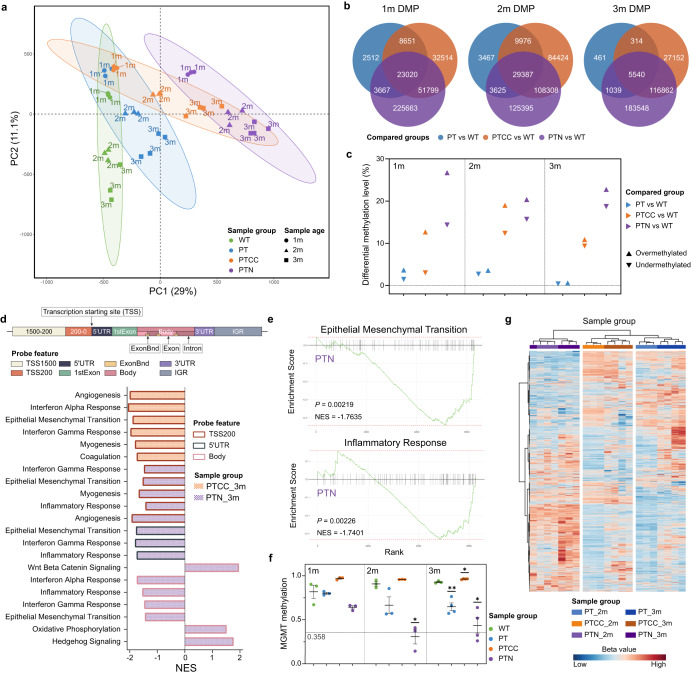
DNA methylome analysis reveals genotype-dependent progressive changes of DNA methylation during gliomagenesis. **a** PCA analysis of 1-, 2- and 3-month-old organoids’ methylome, the ovals indicate 95% confidence ellipse of each genotype. **b** Venn diagrams demonstrate unique and common DMPs in LEGOs at 1, 2, and 3 months compared to age-matched WT. **c** Differential methylation level comparing LEGOs to age-matched WT control. **d** Probe gene feature distribution and GSEA hallmark enrichment of the DMPs located on different gene features (adjusted *P* value < 0.05) in 3-month-old LEGOs. TSS, transcription starting site, UTR, untranslated region, IGR, intergenic region, ExonBnd, exon boundaries. **e** GSEA enrichment plots for the two hallmark gene sets enriched at the 5′UTR region in the 3-month-old PTN organoids. **f** MGMT promoter methylation probability estimation in different organoids. *N* = 3 for all groups of one- and two-month-old organoids and 3-month-old WT organoids, and *N* = 4 for 3-month-old LEGOs. Data are represented as mean ± SEM. *P* values were calculated by Student’s *t* tests comparing LEGOs to age-matched WT, and only significant values are labeled. **P < 0.01, *P < 0.05. **g** Methylation classification heatmap with 8000 GB probes for 2- and 3-month-old LEGOs. See also Supplementary Fig. 3 and Supplementary Table 2.

Tumor-cell DNA methylation was recently used for the molecular classification of brain tumors^6^. However, how different genetic mutations affect the DNA methylation pattern in GBM remains largely unclear. We selected the 1-, 2-, and 3-month-old LEGOs and WT organoids for DNA methylation analysis using an EPIC (850K) DNA methylation array. A principal component analysis (PCA) suggests that the DNA methylome of WT organoids changes gradually over time, indicating a maturation signature of DNA methylation along PC2 (Fig. 3a). Interestingly, the 1-month-old PT and PTCC organoids are similar to the WT organoids (Fig. 3a), indicating that these mutations do not lead to immediate dramatic DNA methylome changes. The PTN organoids differ from PT and PTCC already at 1 month of age (Fig. 3a). Moreover, all LEGOs showed reduced progression along the maturation axis (PC2) compared to WT organoids (Fig. 3a). This also indicates a sign of differentiation blockage, consistent with the scRNA-seq results. On the other hand, PC1 exhibits a gradual but genotype-specific change in DNA methylome (Fig. 3a), suggesting oncogenic mutations induce genotype-specific DNA methylation changes. We then identified differentially methylated probes (DMP) among all groups at different developmental stages and found that DMP numbers were significantly different, with PT organoids showing the lowest and PTN organoids exhibiting the highest (Fig. 3b). Interestingly, the methylation level of the mesenchymal subtype was also shown to be the highest among all three GB subtypes (Supplementary Fig. 3a)^26^. The dynamic changes of DNA methylation in the LEGOs over time demonstrate that the DNA methylome is actively changing during tumor progression (Fig. 3b), including both hypomethylated and hypermethylated probes, particularly during the early developmental stage of brain tumors (Fig. 3c). However, it remains unclear what regulates these dynamic DNA methylome changes during tumor development. We performed a gene set enrichment analysis (GSEA) on the DMPs located on different gene features at various stages. There were no enriched hallmark gene sets in PT, probably due to the low number of DMPs. For the probes located 0-200 bp upstream of the transcription starting site in 3-month-old PTCC organoids, we identified the enrichment of several hallmark gene sets, such as angiogenesis and interferon alpha response (Fig. 3d). The most apparent difference was observed in the PTN group with strong activation of EMT and inflammatory signatures in different gene feature locations at 3 months and in the 5’UTR at 2 months (Fig. 3d, e and Supplementary Fig. 3b), in line with its infiltrative growth pattern in vivo.

MGMT (O^6^-methylguanine-DNA methyltransferase) promoter methylation is associated with better TMZ response in GBM patients^27^. Interestingly, we observed an increased level of MGMT promoter methylation in PTCC organoids compared to PT and PTN organoids (Fig. 3f), which indicates that PTCC organoids may respond better to TMZ treatment than PT and PTN organoids. Moreover, unsupervised cluster analysis demonstrates that different LEGOs can be categorized by human GBM DNA methylation classification probes^26^ (Fig. 3g), indicating a human GBM-like methylation pattern in the LEGO model.

It has been suggested that DNA methylation signatures can be used to determine the cell of origin in human cancers^28^. Our analysis demonstrated that the DNA methylome is dynamic during tumor development and is dependent on the mutation spectrum. Therefore, it is crucial to use stable and mutation-independent DNA methylation patterns as tracers for cancer cell origin. We generated a probe set (Supplementary Table 2) that shows no significant changes among all different groups of organoids. Gene ontology (GO) analysis indicates that these probes are highly enriched for tissue development and differentiation (Supplementary Fig. 3c). This probe set can be further explored as candidates to trace brain tumor origins.

In addition, we also compared the DNA methylome of the iPSC to the 2-month-old organoids in an additional iPSC cell line, and we found that the iPSCs obtained a distinct methylome than the organoids (Supplementary Fig. 3d), which could be attributed to the significance of DNA methylation in the process of cell differentiation. It is also interesting to note that iPSCs from the same genotype do not cluster closely as the LEGOs, suggesting the difference we observed is tumor organoid specific. In this cell line, we also found that the methylation profiles of the WT are distinct from the LEGOs and the PT and PTCC exhibited similarity (Supplementary Fig. 3e).

In conclusion, our results showed that the DNA methylation is largely influenced by the genetic mutations along tumor progression.

### Metabolic reprogramming and metabolic heterogeneity during brain tumor development

**Fig. 4:**
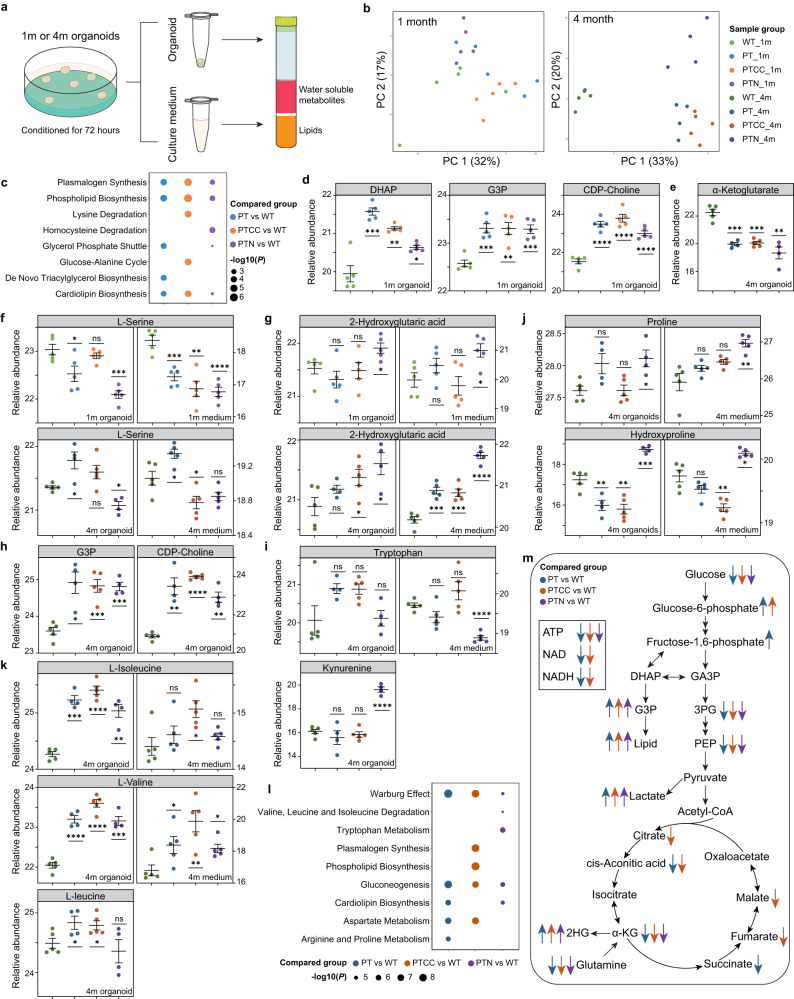
Metabolic reprogramming and metabolic heterogeneity during brain tumor development. **a** Schematic illustration of sample collection and extraction for metabolomic and lipidomic analysis. **b** PCA of 1- and 4-month-old organoid metabolome. **c** Dot plot shows the top five enriched pathways from the quantitative enrichment analysis of 1-month-old LEGOs compared to WT organoids. **d** The relative abundance of lipid metabolism-related metabolites in 1-month-old organoids. **e** The relative abundance of α-ketoglutarate in 4-month-old organoids. **f** The relative abundance of l-serine in 1- and 4-month-old organoids and culture medium. **g** The relative abundance of 2-hydroxyglutaric acid in 1- and 4-month-old organoids and culture medium. **h** The relative abundance of lipid metabolism-related metabolites in 4-month-old organoids. **i** The relative abundance of tryptophan (upper panel) in 4-month-old organoids and culture medium and of the tryptophan metabolite kynurenine (lower panel) in 4-month-old organoids. **j** The relative abundance of the major amino acids constituting collagen in 4-month-old organoids and culture medium. **k** The relative abundance of branched-chain amino acids in 4-month-old organoids and culture medium. **l** Top five enriched pathways from the quantitative enrichment analysis of the metabolites from 4-month-old LEGOs compared to WT organoids. **m** Diagram demonstrating the metabolic changes in LEGOs. GA3P glyceraldehyde-3-phosphate, 3PG 3-phosphoglyceric acid, PEP phosphoenolpyruvic acid. In **d**–**k**, the color of the dots indicates the sample group, data are represented as mean ± SEM; *N* = 4 for 4-month-old PT and PTN organoid samples, and *N* = 5 for the rest of the groups; *P* values were calculated with Student’s *t* tests comparing LEGOs to WT; ****P < 0.0001, ***P < 0.001, **P < 0.01, *P < 0.05, and ns, non-significant. See also Supplementary Fig. 4.

One of the hallmarks of cancer cells is the dysregulation of metabolism^29^. However, it remains unclear how genetic heterogeneity affects the metabolic status of cancer cells. Therefore, we analyzed the intra- and extracellular metabolome of 1- and 4-month-old LEGOs and WT organoids (Fig. 4a).

The metabolome of 1-month-old organoids is largely similar to each other (Fig. 4b and Supplementary Fig. 4a, c). However, enrichment analysis of group-specific changes compared to WT organoids suggests the activation of phospholipid synthesis and glycerol phosphate shuttle in LEGOs (Fig. 4c), indicated by the increase of DHAP (dihydroxyacetone phosphate), G3P (glycerol-3-phosphate) and CDP-choline (Fig. 4d and Supplementary Fig. 4a). This is consistent with our previous finding that GPD1 (glycerol-3-phosphate dehydrogenase 1), which converts DHAP into G3P, is specifically expressed in brain tumor stem cells but not in neural stem cells^30^.

The 4-month-old organoids’ metabolome showed a clear difference between LEGOs and WT organoids (Fig. 4b). PT was similar to PTCC, while PTN was very distinct from the other LEGOs (Fig. 4b). Heatmap analysis of intracellular metabolites demonstrates activation of the glycolysis pathway in LEGOs, indicated by low levels of glucose and glutamine and high levels of lactic acid, further confirmed by the medium metabolite data (Supplementary Fig. 4b, d, e). The TCA (tricarboxylic acid) cycle metabolites (citric acid, aconitic acid, α-ketoglutarate, succinate, fumarate, malate, ATP, NAD) were decreased in LEGOs compared to the WT organoids (Fig. 4e and Supplementary Fig. 4b, f), suggesting a shift toward glycolysis from oxidative phosphorylation, reminiscent of the Warburg effect.

Metabolites are essential substrates of many epigenetic enzymes^31^. We analyzed metabolite changes that may explain the DNA methylome changes in the LEGOs. Serine contributes to methylation via the major methyl group donor S-adenosylmethionine^32^. In 1-month-old organoids, the level of serine in the culture medium was reduced in all LEGOs compared to WT organoids (Fig. 4f and Supplementary Fig. 4a, c), and the intracellular level of serine was most significantly decreased in the PTN organoids (Fig. 4f). In contrast, in 4-month-old LEGOs, the utilization of serine was increased in PTCC and PTN, while decreased in PT (Fig. 4f and Supplementary Fig. 4b, d). This strongly suggests that serine is consumed by all LEGOs and even more by the PTN organoids, which is in line with the observed high levels of hypermethylation in 1- and 3-month-old PTCC and PTN organoids (Fig. 3c). Oxoglutaric acid (α-ketoglutarate, α-KG) is the substrate of many α-KG-dependent dioxygenases, including the DNA demethylation enzymes TET1/2/3 and 2-hydoxyglutaric acid (2-HG) antagonizes the function of α-KG^31^. The level of 2-HG increased in 1-month-old PTN extra- and intracellularly, and accumulated in 4-month-old PTCC and PTN organoids as well as in all LEGO culture media, particularly in the PTN group (Fig. 4g). In contrast, the level of α-KG was depleted in all 4-month-old LEGOs compared to the WT organoids (Fig. 4e). This further explains the dynamic DNA methylation changes in LEGOs and supports the hypermethylation pattern of PTN organoids.

Consistent with the results from 1-month-old organoids, G3P and CDP-choline levels are higher in LEGOs at 4 months of age (Fig. 4h), suggesting the mutant organoids depend on this lipid metabolism pathway. Regarding genotype-specific changes, we found that PTN organoids uniquely upregulate the tryptophan metabolism pathway by consuming and utilizing more tryptophan and producing more kynurenine (Fig. 4i). Kynurenine could be catabolized into NAD to facilitate energy production, cellular proliferation, and immune suppression^33,34^. PTN organoids also have high levels of proline and hydroxyproline in the organoids and culture medium (Fig. 4j). Proline and hydroxyproline are the major amino acid components of collagen proteins^35^. Collagen serves as the scaffold to facilitate glioma cell migration, increase the stiffness of the tumor, and induce an immune suppressive microenvironment^36,37^, and elevated levels of hydroxyproline could be indicative of high collagen turnover. In PTCC organoids, we observed the accumulation of branched-chain amino acids (valine, isoleucine, and leucine) in both the organoids and the medium (Fig. 4k), indicating an abnormal branched-chain amino acid metabolism. Enrichment analysis suggests that the Warburg effect is enriched in all LEGOs, with PTCC particularly showing enrichment of phospholipid biosynthesis, whereas tryptophan metabolism is among the most enriched pathways in PTN organoids (Fig. 4l).

The results above demonstrate distinct metabolic reprogramming events during tumor development (Fig. 4m), and it is evident that genetic mutations determine the metabolic differences in cancer cells. In addition, some metabolic changes may regulate the DNA methylome changes.

### Lipidomics assay uncovers glycerol lipid metabolism being a hallmark of GBM

**Fig. 5:**
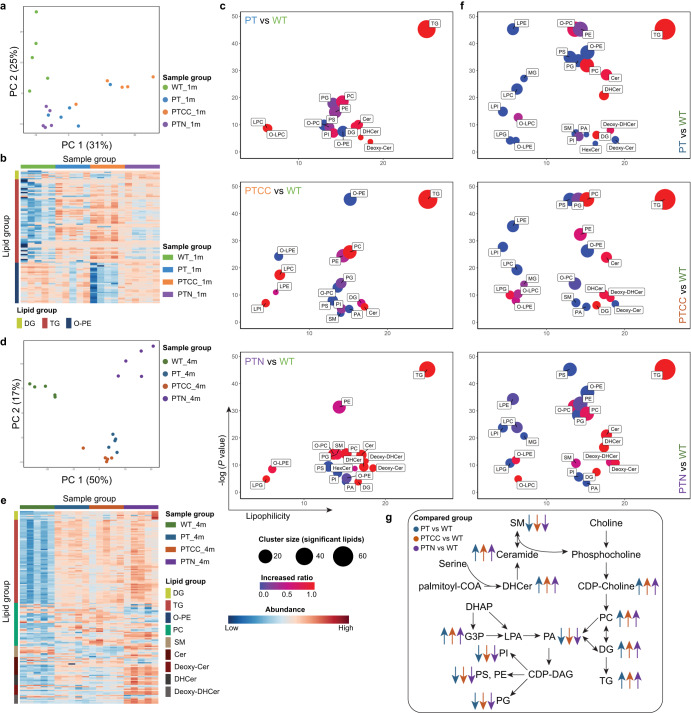
Lipidomics assay uncovers glycerol lipid metabolism being a hallmark of GBM. **a** PCA of the 1-month-old organoid lipidome (**b**). Abundance heatmap of different lipids in 1-month-old organoids. **c** Enrichment plot for different lipid groups in 1-month-old organoids. **d** PCA of 4-month-old organoid lipidome. **e** Abundance heatmap of different lipids in 4-month-old organoids. **f** Enrichment plot for different lipid groups in 4-month-old organoids. **g** Diagram demonstrating the lipidomic changes in the LEGOs compared to WT organoids. In **c** and **f**, the cluster size indicates the number of significantly changed lipids in the group comparison. The increased ratio was calculated by dividing the number of significantly increased lipids by the total number of significantly changed lipids. See also Supplementary Fig. 5.

The metabolomic analysis identified that the metabolites (DHAP, G3P, CDP-choline) in phospholipid biosynthesis are strongly associated with GBM development. We therefore performed lipidomic analysis using the same experimental setup shown in Fig. 4a. The PCA analysis of 1-month-old organoids shows that the lipidomes of LEGOs are different from WT organoids (Fig. 5a). This was unlike the metabolome and methylome results, suggesting that lipidome reprogramming is the pioneering event upon the loss of tumor suppressors. The heatmap of lipid species indicates both DG (diacylglycerols) and TG (triacylglycerols) upregulation in all mutant groups (Fig. 5b), which further illustrates the consequence of increased DHAP and G3P. This was further confirmed by enrichment analysis showing that TGs are the most significantly enriched lipid species in all LEGOs (Fig. 5c). In addition, a decrease in ether-linked phosphatidylethanolamine (O-PE) was observed in the PTCC organoids (Fig. 5b, c).

We next analyzed the lipidome of 4-month-old organoids. PCA was similar to the 4-month-old metabolome PCA, with the leading principal component (PC1) separating the LEGOs from WT and the second principal component (PC2) distinguishing PTN from PT and PTCC (Fig. 5d). DGs, TGs, and phosphatidylcholine (PC) were significantly increased in all mutant organoids, particularly in PTN (Fig. 5e, f). This, together with the increase of G3P, DHAP, and CDP-choline, as described above, highlights the importance of TG and choline metabolism in GBM (Fig. 5g). On the other hand, the structural phospholipids (such as PG, PI, PS, PE, and O-PE) are decreased in mutant organoids (Supplementary Fig. 5a). It is likely that the increased production of DG, TG, and PC in LEGOs leads to decreased structural phospholipids as these lipids are derived from the same precursor, G3P. Ceramide production could be activated under stress conditions by hydrolyzing sphingomyelin (SM)^38^. Consistently, we observed a higher amount of SM in WT, and abundant ceramides and CDP-choline in all LEGO groups (Figs. 5e–g and 4h), suggesting augmented activation of SM hydrolysis. PTN exhibited significantly higher ceramide expression than all other groups (Fig. 5e), implying a unique mechanism enhancing ceramide synthesis upon loss of *NF1*. It was shown that the tryptophan metabolite kynurenine can directly bind and activate the aryl hydrocarbon receptor (AHR)^34,39^, and the activation of AHR elevates the synthesis of ceramides^40,41^. Altogether, the lipidome analysis identified that lipid reprogramming is a pioneering event during gliomagenesis, and glycerol lipid metabolism is a hallmark of GBM (Fig. 5g).

### Proteomic/phospho-proteomic analysis identifies actionable targets and pathways for the genotype-based treatment of GBM

**Fig. 6:**
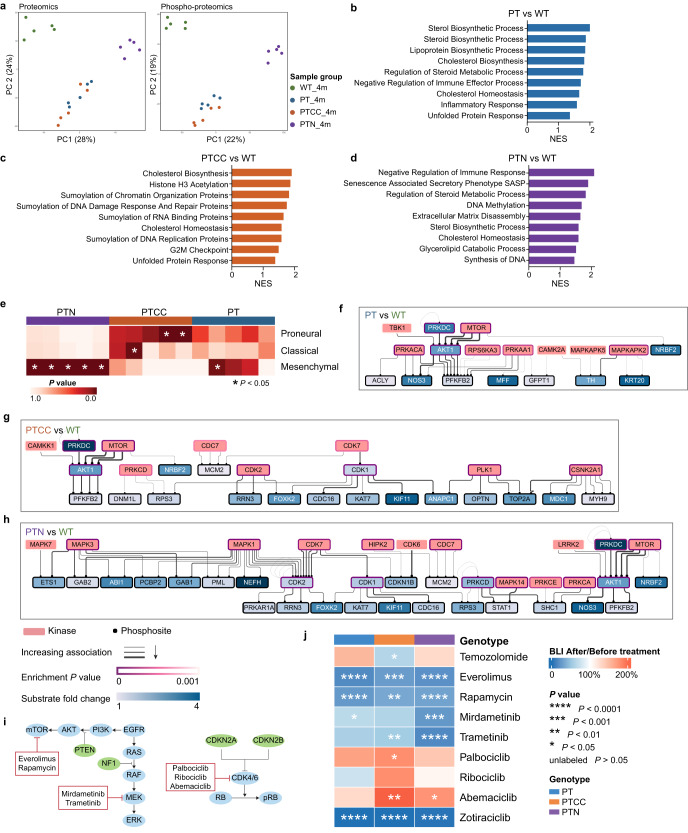
Proteomic/phospho-proteomic analysis identifies actionable targets and pathways for the genotype-based treatment of GBM. **a** PCA of proteomics and phospho-proteomics of 4-month-old organoids. Representative significantly enriched pathways or terms in PT (**b**), PTCC (**c**), and PTN (**d**) compared to WT identified by GSEA. **e** Heatmap showing the enrichment of GBM subtype signatures^42^ in different LEGOs. Enriched kinases together with their substrates in PT (**f**), PTCC (**g**), and PTN (**h**) compared to WT. **i** Illustration of selected drugs and their targets. **j** Treatment outcome for the proof-of-principle drug tests. *N* = 3 for TMZ treatment groups, *N* = 4 for kinase inhibitor treatments. *P* values were calculated with paired Student’s t-tests comparing signals measured after treatments to before treatments See also Supplementary Fig. 6, Supplementary Table 3, 4, and 5.

To search for possible genotype-specific drug targets using the LEGO models, we next performed proteomic and phospho-proteomic analyses on 4-month-old organoids. Proteome and phospho-proteome PCA plots exhibit high similarity to the metabolome and lipidome, with a distinct difference between LEGOs and WT organoids, and PTN shows a more distinct proteome/phospho-proteome profile compared to PT and PTCC (Fig. 6a). However, phospho-proteome provided a better separation between PT and PTCC (Fig. 6a). GSEA analysis of differentially expressed proteins identifies processes involved in LEGO development (Supplementary Table 3). In particular, the cholesterol and lipid pathways are enriched in all LEGOs (Fig. 6b–d), consistent with the metabolomic and lipidomic data. The G2M checkpoint and related stress and mitosis pathways are enriched in PTCC (Fig. 6c), indicating elevated mitosis as a result of *CDKN2A/2B* deletion. PTN organoids are enriched for negative regulation of immune response and SASP (senescence-associated secretory phenotypes) (Fig. 6d and Supplementary Fig. 6a). Additionally, signatures associated with DNA methylation, extracellular matrix disassembly, as well as several collagen proteins are highly enriched in PTN (Fig. 6d and Supplementary Fig. 6b). This is concordant with the observed DNA methylome changes, the in vivo infiltrative phenotype of PTN tumors, and the proline/hydroxyproline enrichment in PTN metabolome, respectively.

RNA expression has been used for the molecular classification of GBM, but little is known about whether using protein expression as the classifier will yield similar results. We analyzed our proteome data using established tumor-cell-specific RNA signatures^42^ and found that mesenchymal signatures are highly enriched in the PTN organoids (Fig. 6e and Supplementary Fig. 6c). This result, together with the other omics analyses, firmly confirms that the PTN organoid resembles the mesenchymal subtype of GBM. Furthermore, we found that the expression of MGMT protein is high in the PTN group, and expression of IDH1 is increased in PT and PTCC compared to WT, concordant with the methylation changes (Supplementary Fig. 6d).

To identify possible actionable targets in different subgroups of LEGOs, we utilized a drug-gene interaction database^43^ to identify druggable targets for LEGOs (Supplementary Table 4). Collectively, enzymes involved in lipid metabolism enzymes, such as MGLL (monoglyceride lipase), and FDFT1 (farnesyl-diphosphate farnesyltransferase 1), could be potential targets for all LEGOs (Supplementary Fig. 6e); this is in line with the activation of lipid metabolism in LEGOs. DNMT3A (DNA methyltransferase 3A), SPTLC2 (serine palmitoyltransferase 2), and cholinesterase (BCHE) could be interesting targets for PTN (Supplementary Fig. 6e).

The phospho-proteomic data allow the prediction of possible kinases involved in tumor progression. We used a kinase-target interaction database^44^ and Kinase Enrichment Analysis^45^ to identify the upstream kinases of the phosphorylated sites (Supplementary Table 5). PT organoids showed activation of AKT1 and mTOR, due to the mutation of *PTEN* (Fig. 6f). Surprisingly, the PTCC organoid phospho-proteomic data did not show enrichment of CDK4/6, which are classic substrate kinases of CDKN2A/2B. Instead, CDK1/2/7 were activated in addition to mTOR and AKT1 (Fig. 6g). In addition to AKT1 and mTOR, MAPK1, MAPK3 and CDK7 were upregulated due to *NF1* mutation in PTN organoids (Fig. 6h). Using luciferase as a readout for tumor cells in the LEGO model, we found that PTCC LEGO is more sensitive than the other two to TMZ (Fig. 6j), in line with a higher MGMT promoter methylation status. the mTOR inhibitors were effective in all LEGOs (Fig. 6i, j, Supplementary Fig. 6f, 6g), consistent with the kinase enrichment analysis and the previous published results that *PI3K* mutant GBOs are sensitive to Everolimus treatment^46^. In contrast, CDK4/6 inhibitors exhibited no growth inhibition in all LEGOs (Fig. 6j), again concordant with the kinase enrichment analysis, which indicated that CDK4/6 were either not enriched or only showed low enrichment compared to other CDKs. On the other hand, MEK1/2 inhibitors were effective in all groups but most effective in PTN organoids, likely because of the enhanced activation of MAPKs (Fig. 6j). The sensitivity of MEK1/2 inhibitor in PTN organoids was consistent with that have been published in *NF1* mutant GBOs^46^. Combination therapy using mTOR and MEK inhibitors shows the most effective inhibition of PT and PTN growth, while CDK4/6 inhibitors compromise mTOR inhibitor effects in PTCC organoids (Supplementary Fig. 6 h), suggesting that CDK4/6 inhibitors should be carefully examined before being considered for treating GBM patients with *CDKN2A/2B* mutations. Moreover, we treated the LEGOs with the CDK inhibitor Zotiraciclib targeting CDK1/2, which was highly activated in all the mutant organoids, and observed that all LEGOs were highly sensitive to Zotiraciclib treatment (Fig. 6j), suggesting that CDK1/2 are valuable therapeutic targets in GBM.

To sum up, the proteomics analysis demonstrated significant consistency with other omics analyses, and the proof-of-principle drug test indicated that LEGOs are promising tools for assessing drug efficacy.

### LEGO-based drug screening identifies new drug candidates for GBM therapy

**Fig. 7:**
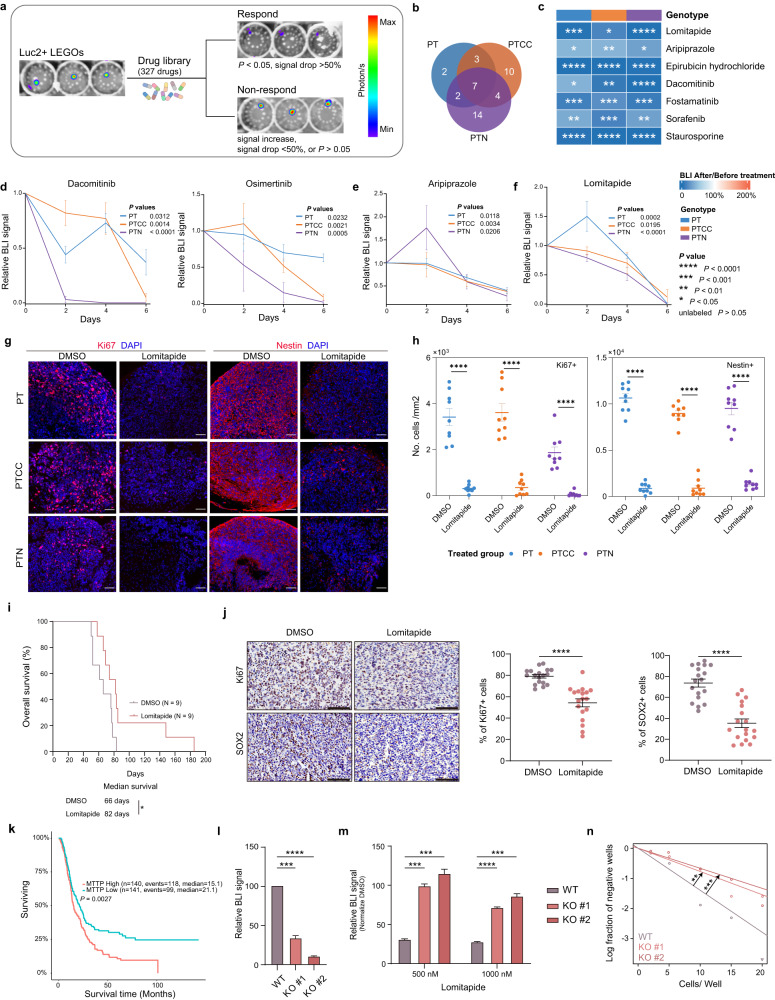
LEGO organoids respond to drugs that target mutation-specific mechanisms. **a** Illustration of BLI-based drug screen. **b** Venn diagrams demonstrating effective drug distribution in different LEGOs. **c** Treatment outcome for drugs effective in all LEGOs. *N* = 3 for each group. Cell viability tracing with BLI signal in LEGOs treated with Dacomitinib and Osimertinib (**d**), Aripiprazole (**e**), and Lomitapide (**f**). **g** Representative Ki67 and Nestin staining on LEGOs treated with DMSO or Lomitapide. Scale bar, 50 μm. **h** Quantification of Ki67+ and Nestin+ cells treated with Lomitapide, *N* = 9 sections for each group. **i** Overall survival of tumor-bearing mice treated with DMSO or Lomitapide. *P* value was calculated with Log rank test. **j** Representative staining images of tumor areas in mice treated with DMSO or Lomitapide. Quantification of Ki67-positive and SOX2-positive cells is provided on the right. *N* = 6 section for each group. Scale bar: 100 µm. **k** MTTP expression on GBM patient survival from an external data set^48^, *P* value was calculated with Log rank test. **l** Relative growth of *Mttp* WT or KO *Pten/Trp53* KO mouse BTSCs after 48 h of culture. **m** Relative growth of *Mttp* WT or KO *Pten/Trp53* KO mouse BTSCs after 48 hours of treatment with 500 nM or 1000 nM Lomitapide. **n** Limiting dilution assay for *Mttp* WT or KO *Pten/Trp53* KO mouse BTSCs. In c–f, *P* values were calculated with paired Student’s t-tests comparing signals measured after treatments to before treatments; ****P < 0.0001, ***P < 0.001, **P < 0.01, *P < 0.05. In **d**–**f**, **h**, **j**, **l**, and **m**, data are represented as mean ± SEM. See also Supplementary Fig. S7, Supplementary Table 6, and Table 7.

With the goal of generating a genotype-based drug reference map and possibly identifying new treatment strategies for GBM, we performed a drug screen on 327 drugs containing FDA-approved drugs that could penetrate through the blood-brain barrier (Fig. 7a and Supplementary Table 6). All LEGO cells were engineered to express luciferase, and the bioluminescence signal was used as a readout of cell numbers in the LEGOs. To select effective drugs, among the drugs that resulted in significant inhibition of bioluminescence signal (*P* < 0.05), we only selected drugs that resulted in 50% inhibition of bioluminescence signal as positive candidates. With this screen, we identified 42 drugs with therapeutic effects; seven acted on all three genotypes, and the rest only worked on specific genotypes (Fig. 7b, c and Supplementary Fig. 7a). We found that EGFR inhibitors Dacomitinib and Osimertinib inhibit LEGO growth in all genotypes, with a particularly strong effect on the PTN organoid (Fig. 7c, d and Supplementary Fig. 7a), suggesting a strategy of patient enrollment for clinical trials for testing EGFR inhibitors. The Syk (spleen tyrosine kinase) inhibitor Fostamatinib inhibits all LEGOs, suggesting that Syk signaling is essential for GBM progression (Fig. 7c). Interestingly, we also found that the schizophrenia drug Aripiprazole also inhibits tumor growth in all LEGOs (Fig. 7c, e), which implies an alteration of dopamine signaling in GBM. Then, we performed IC50 tests on three specific drugs — Aripiprazole, Osimertinib, and Lomitapide. The results of these tests demonstrated that the IC50 values for LEGOs (Supplementary Fig. 7b) were in line with the findings from the initial drug screen conducted at the 10 µM concentration. Subsequently, we applied the maximum IC50 values obtained from LEGOs to treat WT organoids (Supplementary Fig. 7c). Our findings demonstrated that these drugs exhibited specific efficacy on LEGOs while having no discernible impact on WT organoids.

Since our multi-omics analysis showed that lipid metabolism plays an important role in tumor progression, we focused further on the analysis of Lomitapide, an inhibitor of microsomal triglyceride-transfer protein (MTTP), which could inhibit tumor growth in all LEGOs (Fig. 7c, f). MTTP is a lipid transfer protein and is essential for the regulation of lipid metabolism. This is in line with our discovery that glycerol lipid metabolism is a hallmark of GBM metabolism. In the Lomitapide-treated LEGOs, we found a striking reduction in the number of proliferating cells and stem cells (Fig. 7g, h). We then conducted additional experiments to assess the impact of Lomitipade in vivo. We initiated a xenograft model by injecting *Pten/Trp53* KO mouse BTSCs^47^ into the brains of C57/BL6N mice. Once tumors were established, they were randomly assigned to two groups and treated with either DMSO or Lomitapide. The survival analysis revealed a significant extension of overall survival in the group treated with the Lomitapide (Fig. 7i and Supplementary Table 7). Furthermore, the staining demonstrated the elimination of not only proliferating cells but also a significant proportion of stem cells (Fig. 7j), aligning with the results observed in LEGOs.

Expression of Mttp has highly enriched in our previous ribosome RNA-sequencing analysis by comparing mouse neural stem cells (NSCs) and brain tumor stem cells (BTSCs), its expression is also highly enriched in tumor-bearing mice after TMZ treatment (Supplementary Fig. 7d)^30^. High expression of MTTP also shows a worse prognosis in GBM patient (Fig. 7k)^48^, and single-cell analysis in published data sets^9^ suggest MTTP is more expressed in stem cell or mesenchymal subtype of tumor cells (Supplementary Fig. 7e), which is consistent with our previous observation of activation of glycerol metabolism in BTSCs^30^.

To further confirm that Lomitapide exerts its effects through MTTP, we performed *Mttp* knockout in the *Pten/Trp53* KO mouse BTSCs (Supplementary Fig. 7f). The results indicated that *Mttp* knockout cells exhibited slower growth compared to WT cells (Fig. 7l). Furthermore, they displayed increased resistance to Lomitapide treatment (Fig. 7m) and were less likely to form tumor spheres due to the elimination of stem cells (Fig. 7n). Based on these compelling results, we believe that Lomitapide warrants further investigation as a potential treatment for GBM.

Additionally, to rule out the possibility that the drug screen results are specific to the GFP+ iPSC, we performed bulk RNA sequencing on 2-month-old organoids derived from two iPSC lines. The analysis revealed significant differences between the WT and knockout organoids, with a clear separation of PTN clearly distinguishing itself from PT and PTCC (Supplementary Fig. 7g). In addition, our GSEA enrichment analysis unveiled enriched pathways related to lipid metabolism in PT, PTCC, and PTN when compared to WT. Furthermore, PTN displayed significant enrichment in pathways associated with “Negative regulation of immune response” and “extracellular matrix disassembly” (Supplementary Fig. 7h). Further, as a proof of concept, we conducted a drug screening test using LEGOs derived from the additional iPSC cell line. Our findings mirrored those observed in the original cell line, with mTOR inhibitors demonstrating consistent efficacy across all three genotypes, CDK4/6 inhibitors exhibiting no discernible effect, and CDK1/2 inhibitors showing notable effectiveness (Supplementary Fig. 7i). Particularly, MEK1/2 inhibitors emerged as the most efficient in PTN organoids. Moreover, the drug identified through multi-omics analysis and prior drug screening, Lomitapide, exhibited favorable results in LEGOs derived from the new cell line (Supplementary Fig. 7i).

Complete information on treated drugs and outcomes can be found in Supplementary Table 6. Noteworthily, some drugs that exhibited therapeutic effects on one genotype may promote the growth of another, which further highlights the importance of genetic background in directing treatment options. This genotype-based drug reference provides a basis for the personalized treatment of GBM patients.

## Discussion

Our temporal multi-omics analysis (scRNA-seq, DNA methylome, Metabolome/Lipidome, and Proteome/Phospho-proteome) covers essential molecular layers of the cancer cell molecular network. This allows us to discover genotype-specific molecular changes during tumor development. In Supplementary Table 8, we summarized all major molecular milestones during GBM development and divided them into shared and genotype-specific milestones. We also list milestones that can be validated by analysis of different molecular layers. i.e., early changes during GBM development include the increase of stem cell frequency and attenuation of neural differentiation. This is accompanied by metabolite changes, which can also influence epigenetic modifications like DNA methylation. Coherently, active DNA methylation changes during early tumor development shown by methylation array data, elevated DNA methylation pathway activity, and low IDH1 expression presented by proteomics data support hypermethylation in PTN, which could be further confirmed by the decrease of a-KG and increase of 2-HG in the metabolomic assay. The increase of phospholipid metabolism is an early event, and this change persists with brain tumor development. Notable genotype-specific features include a WNT activation in PTCC organoids, ectopic expression of the HOX gene cluster, and mesenchymal signature in PTN organoids. The MGMT promoter is methylated in PTCC organoids, and we show that PTCC organoids are sensitive to TMZ. It is also important to note that the LEGOs are primarily similar to the WT organoids at the 1-month-old, highlighting that most of the oncogenic changes occurred during tumor organoid development, not at the iPSC stage.

One fundamental question in cancer biology is which features of cancer cells are determined by genetic and non-genetic heterogeneity, respectively. This could not be investigated so far due to the lack of proper models. The genetically defined LEGOs are initially derived from the exact iPSC clone providing an ideal tool to assess the contribution of genetic heterogeneity to intratumoral heterogeneity quantitatively. In our analysis, *CDKN2A/2B* mutation in *PTEN* and *TP53* deletion background further push the development of PT organoids in a similar direction, suggesting these mutations work together and drive similar cancer phenotypes. However, the *NF1* mutation dramatically reprograms the cancer cell phenotypes across all molecular layers, which will be discussed below. Therefore, the LEGO model can serve as genetic building blocks of the cancer genome, which can be further expanded and used to analyze the interaction between cancer genetic and non-genetic heterogeneity. Combining LEGOs to generate fully customized genetically heterogenous organoids is also straightforward. The scRNA results also demonstrated that genetic mutations have mutation-specific influences on cell phenotypes. Although the stem cell differentiation hierarchy is largely maintained in all LEGOS. The cellular composition and molecular phenotype of the lineages in different LEGOs are different from each other. This is critical information for future interpretation of scRNA-seq results of human GBM patient tissues; the contribution to cellular heterogeneity from genetic and non-genetic factors must be clearly demonstrated. Therefore, obtaining mutation information and considering the genetic heterogeneity within different cell clusters is essential before claiming they may represent different cell states^9^.

Another striking observation in our multi-omics analysis is the activation of phospholipid metabolism throughout LEGO development. Interestingly, this activation is already noticeable in 1-month-old LEGOs, supported by increased DHAP and G3P. DHAP is the intermediate metabolite of glycolysis and can be converted by GPD1 into G3P, the primary precursor for lipid metabolism. We have shown before that GPD1 is induced explicitly in brain tumor stem cells during brain tumor development and blocking GPD1 alters tumor lipid metabolism and prolongs the survival of brain tumor-bearing animals^30^. The increase of DHAP, G3P, and CDP-choline in the metabolomic analysis and the increase of DG, TG, and PC in the lipidomic analysis in LEGOs demonstrate that lipid metabolism, particularly the glycerophospholipid metabolism, is activated during brain tumor development. This is in line with the clinical observation that the glycerol level in GBM patients is much higher in tumors compared to normal tissue in the tumor periphery^49^. It was also reported that brain metastasis also upregulates lipid metabolism^50,51^, indicating an adaptation of cancer cells to the lipid-deprived brain environment^50,52^. For this purpose, brain tumor cells upregulate GPD1 to switch the metabolic flow to lipid metabolism by making use of the glycolysis metabolite DHAP, which was also reported to be the only sensor metabolite of the mTOR pathway in glycolysis^53^. More importantly, we also discovered that MTTP inhibitor Lomitapide efficiently blocks LEGO growth, providing another attractive target, and the drug should be further investigated. Lipid metabolism is likely an emerging hallmark of brain cancers that should be further investigated.

Major mutations that drive human GBM have been identified via genomic sequencing^4,5^. Interestingly, the major molecular subtypes of human GBM are defined primarily via RNA expression or DNA methylation pattern^8,42,54^, and there is no strong correlation between genetic mutation and molecular subtypes. *NF1* mutation is highly enriched in the mesenchymal subtype, whereas *TP53*, *PTEN*, and *CDKN2A/2B* inactivation were not enriched in particular subtypes^4^. Inactivation of *Nf1* and *Trp53* leads to brain tumor formation in a mouse model^55^. However, whether *NF1* mutation drives mesenchymal GBM remains not experimentally confirmed. Here we showed that *NF1* mutant organoids have many unique features compared to other LEGOs. The PTN xenograft shows a rather infiltrative growth pattern and high angiogenesis, and scRNA-seq identified a mesenchymal cell cluster with increased expression of collagen genes. PTN also produces the immunosuppressant kynurenine and has high levels of proline and hydroxyproline, which support the high collagen level. Moreover, PTNs are not sensitive to TMZ treatment because of lacking MGMT methylation and increased expression of MGMT protein. All these factors fit the mesenchymal features of human GBM^23^ and confirm that *NF1* mutation drives the mesenchymal features in human GBM. The significant differences between PTN tumors and PT/PTCC tumors suggest that the *NF1* mutant GBM is a unique subgroup of GBM, which should be studied and treated differently.

Compared to several previously published GBM organoid models, LEGO exhibited several advantages. For example, neoCOR is well-suited for the study of invasion and the interaction between tumor and normal cells, but it may not fully encompass the common molecular subtypes of GBM due to its reliance on typical GBM driver mutations. Additionally, the limited representation and characterization of tumor cells in this model makes large-scale omics analyses challenging^7^. Similarly, the glioblastoma model using human cerebral organoids^8^ is valuable for investigating interactions between tumor and normal cells but may not provide a comprehensive representation of the diverse molecular subtypes of GBM.

The LEGOs present a notable advantage in that they originate from a particular iPSC clone with precisely defined mutations. This distinctive attribute empowers us to quantitatively investigate how genetic diversity contributes to phenotypic heterogeneity, a facet that was challenging to assess in prior models.

The LEGO model analysis demonstrates that genetic mutations determine major molecular consequences. Therefore, the realization of personalized treatment of human GBM requires knowledge of genotype-specific drug sensitivity information. The LEGO model comprehensively encompasses the primary GBM subtypes and is well-suited for establishing links between tumor genotype and drug response. Our preliminary treatment of LEGOs demonstrates that different LEGOs respond differently to drug treatments. This set the foundation for using LEGO-like models to study human cancer heterogeneity. The results obtained from the LEGOs show an excellent correlation across different molecular layers, including drug responses. The MGMT promoter was found to be highly methylated in PTCC organoids, and the PTCC organoids respond better to TMZ treatment. In particular, it is unexpected that the PTCC organoid do not respond to CDK4/6 inhibitors and our phospho-proteome results suggest CDK4/6 are not activated in the PTCC organoids. This raises concern about using a *CDKN2A/2B* mutation as a selection criterion for CDK4/6 inhibitors. The drug screen we performed also provided precious information on a genotype-based drug sensitivity map, which can be used for drug candidate selections on personalized treatment GBM clinical trials.

It is also important to mention that the LEGO models are based on iPSCs, which can be easily shared with the research community for comparison, validation, and improvement. The other models are limited either with limited patient material access or the requirement of new tumor induction, which limits the potential for cross-lab comparison and validation.

The following steps will further expand the LEGO genotypes and assemble different LEGOs to build fully customized, genetically heterogenous organoids, that can be used to investigate clonal evolution, cell competition, clonal interactions, and combination therapies. Moreover, adding relevant tumor stromal cells like microglia and T cells in a controlled way will also be interesting, as it will allow investigations into how genetic heterogeneity determines immune cell behavior.

## Methods

### Genome editing of iPSCs

Human induced pluripotent stem cells (iPSC) with mEGFP inserted at the safe harbor locus AAVS1 under CAGGS promoter were purchased from Coriell Institute (New Jersey, USA, Cat#AICS-0036-006; RRID: CVCL_JM19). Another human iPSC line was provided by RUCDR Infinite Biologics (RUID: 06C53141). All the iPSCs were cultured in Matrigel (Corning, New York, USA, Cat#354277) coated plates, fed with mTeSR Plus medium (Stemcell Technologies, Vancouver, Canada, Cat#100-0276) every other day at 37 °C incubators supplied with 5% CO_2_. The cells were passaged with ReleSR (Stemcell Technologies, Cat#05872) as small colonies after reaching 70–80% confluency. 3 μM of CHIR99021 (Tocris Bioscience, Minneapolis, USA, Cat#4423) and 1 μM of PD0325901 (Selleckchem, Houston, USA, Cat#S1036)^19^ were added to the culture medium of PTN iPSCs. The cultures were regularly tested for mycoplasma contamination.

The gRNAs targeting respective tumor suppressor genes were inserted into modified pX330 plasmids^56^ containing the puromycin-resistant gene. The electroporation was conducted with Neon™ Transfection System (Thermo Fisher Scientific, Massachusetts, USA). Briefly, cells were harvested by four minutes of Accutase (Sigma-Aldrich, Missouri, USA) treatment at 37 °C and resuspended with R resuspension buffer containing 15 μg gRNA expression vectors. The electroporation was conducted for two pulses with 1200 V, 20 ms. The electroporated cells were cultured in mTeSR Plus medium containing ROCK inhibitor (Stemcell Technologies, Cat#72304) for 24 hours after the electroporation. Puromycin (2 μg/mL) was added to the culture medium and refreshed every 12 hours for two days. The cells were then seeded at a density of 50 ~ 100 cells per 10 cm dish and expanded for ten days. The single cell colonies were screened by T7 Endonuclease I (T7E1, New England Biolabs, Ipswich, USA, Cat#M0302L) assay, and the Sanger sequencing (Eurofins) results were analyzed with the TIDE webtool^57^. The A-tailing PCR products from the candidate clones were cloned into the pGEM-T vector (Promega, Madison, USA, Cat#A3600) and sequenced. Then two clones from each mutation combination were selected and further validated by western blotting. In brief, the protein lysis from the iPSCs was electrophoresed and transferred onto 0.2 μm PVDF membranes. The membranes were blocked with 5% non-fat milk for one hour at room temperature (RT) and incubated with primary antibodies overnight at 4 °C with shaking. The next day, the membranes were incubated with respective horseradish peroxidase (HRP) conjugated secondary antibodies for two hours and imaged using ChemiDoc (Bio-Rad, California, USA) after reacting with HRP substrate. gRNA sequences (5’ to 3’): *PTEN*, CAGTTTGTGGTCTGCCAGCT, *TP53*, GCAGTCACAGCACATGACGG, *CDKN2A*, GATGATGGGCAGCGCCCGAG, *CDKN2B*, CTGGCCAGCGCCGCGGCGCG, *NF1*, CCAGGATATATCCAAAGACG. Antibody dilutions: PTEN (Cell Signaling Technology, Massachusetts, USA, Cat#9559 L, RRID: AB_390810), 1:1000; P53 (Thermo Fisher, Cat#MA512557, AB_10989883), 1:1000; P15/P16 (Santa Cruz, Texas, USA, Cat#sc-377412), 1:50; NF1 (DKFZ, Heidelberg, Germany, Cat#DKFZ-NF1-146/29/25^58^), 1:4; GAPDH (Cell Signaling Technology, Cat#2118 L, Cat#2118 L), 1:2000; β-Tubulin (Cell Signaling Technology, Cat#2128 s, Cat#2128 s), 1:2000.

### Organoid culture

The WT, PT, PTCC, and PTN organoids were generated following previously described protocols^20^^,^^59^^,60^ with minor adaptions. Briefly, on day 0, the iPSCs were dissociated into single cells as described above and 12,000 cells were seeded each well of 96-well ultra-low attachment plates (Corning, Cat#7007) containing the following medium, 80% DMEM/F12 (v/v, Gibco, Montana, USA, Cat#11330032), 20% KOSR (v/v, Gibco, Cat#10828-028), 3% ES-qualified fetal bovine serum (v/v, FBS, Gibco, Cat#10270106), 1% GlutaMAX (v/v, Gibco, Cat#35050038), 1% MEM-NEAA (v/v, Sigma-Aldrich, Cat#11140050) and 0.7% 2-Mercaptoethanol (v/v) supplied with 50 μM of ROCK inhibitor and 6 ng/mL of bFGF (Peprotech, New Jersey, USA, Cat#100-18B). The medium was refreshed on day 3. On day 5, the culture medium was replaced with DMEM/F12 containing 1% N2 (v/v, Gibco, Cat#17502048), 1% GlutaMAX (v/v), 1% MEM-NEAA (v/v), and 1 μg/mL heparin (Sigma-Aldrich, Cat#H3149) and cultured for four days. The Ebs were then embedded in Matrigel and cultured in the following medium, 50% DMEM/F12 (v/v), 50% Neurobasal (v/v, Gibco, Cat#21103049), 0.5% N2 (v/v), 2% B27 without Vitamin A (v/v, Gibco, Cat#12587010), 0.025% insulin (v/v, Sigma-Aldrich, Cat#I9278), 0.35% 2-Mercaptoethanol (v/v,), 1% GlutaMAX (v/v), 0.5% MEM-NEAA (v/v), and 1% Penicillin/Streptomycin (v/v) for four additional days in 6-well ultra-low attachment plates (Corning, Cat#3473). Thereafter, the organoids were cultured on orbital shakers in culture medium containing 50% DMEM/F12 (v/v), 50% Neurobasal (v/v), 0.5% N2 (v/v), 2% B27 (v/v, Gibco, Cat#17504044), 0.025% insulin (v/v), 0.35% 2-Mercaptoethanol (v/v), 1% GlutaMAX (v/v), 0.5% MEM-NEAA (v/v), 1% Antibiotic-Antimycotic (v/v, Gibco, Cat#15240096) and 0.4 mM l- Ascorbic Acid (Sigma-Aldrich, Cat#A4544). The medium was exchanged every two to three days until sample collection. 3 μM of CHIR99021 and 1 μM of PD0325901^19^ were added to the PTN culture for the first 5 days. Widefield images for the organoids were taken by the Cell Observer (Zeiss, California, USA).

### Luciferase labeling of iPSCs

HEK293T cells were cultured with IMDM (Gibco, Cat#31980030) supplied with 10% FBS (v/v, ATCC, Cat#30-2020) at 37 °C incubators supplied with 5% CO_2_ and passaged with Trypsin/EDTA (Gibco, Cat#15400054). For lentivirus production, 5 ×10^6^ cells were seeded in 10 cm dishes and co-transfected with 2 μg envelope plasmid pMD2.G, 2 μg packaging plasmid psPAX2, and 4 μg luciferase (Luc2) expressing vector pHHLVX-EF1α-Luc2-puro the next day. DNA vectors were mixed with OptiMEM (Gibco, Cat#31985062) to a total volume of 250 μL, and 3x DNA volume polyethyleneimine (PEI, 1 mg/mL) was diluted in OptiMEM to a total volume of 250 μL, respectively. The two mixtures were then combined, thoroughly mixed, and incubated at RT for 15 min before being dropwise applied to the HEK293T cells. The virus particles were collected 24 hours and 48 hours after transfection and concentrated with Lenti-X™ Concentrator (Takara, California, USA, Cat#631232). The pellets were then resuspended with PBS, aliquoted, and stored at −80 °C. The iPSCs were infected with the lentivirus, and positive clones were selected by bioluminescence imaging (BLI) with IVIS Lumina II In Vivo Imaging system (PerkinElmer, Waltham, USA). Ten Luc2 positive clones were pooled to maximize the labeling rate and minimize the colony effect.

### Sample collection and cryosection

The organoids were fixed in 4% PFA for 20 min to 1 hour at 4 °C and emerged in 30% sucrose (w/v) overnight at 4 °C to dehydrate the tissue. The next day, the organoids were embedded in Gelatin/Sucrose solution and froze on dry ice. Gelatin/Sucrose solution was prepared by dissolving 7.5% gelatin (w/v) in 10% sucrose (w/v) at 37 °C. The embedded samples were stored in sealed plastic bags in a −80 °C freezer. Sections were collected and dried for one hour at RT before storing at −80 °C.

### Hematoxylin and Eosin staining

The paraffin sections were deparaffined with the following procedure: 2 × 5 min Xylene, 2 × 5 min 100% Ethanol, 2 × 5 min 95% Ethanol, and 5 min 70% Ethanol. The sections (cryosections or deparaffined sections) were rehydrated in ddH_2_O for 5 min, stained in Hematoxylin for 1.5 min, and rinsed for 5 min under running tape water. 0.1% Eosin was applied for 1.5 min, washed by dipping in water, and differentiated in 70% Ethanol for 3 min. Dehydration was done with the following changing of buffers: 3 min 85% Ethanol, 2 × 5 min 100% Ethanol, 2 × 5 min Xylene. The sections were mounted with Eukitt and imaged with Axioscan (Zeiss) or Tissue FAXS Plus (Tissue Gnostics, California, USA).

### Immunofluorescence and immunohistochemistry

For Immunofluorescence (IF) staining, the sections were incubated with primary antibodies overnight at 4 °C after deparaffinization (for paraffin sections only), rehydration, antigen retrieval, and blocking. The sections were then incubated with respective secondary antibodies conjugated with AlexaFluor (Thermo Fisher Scientific, AF488, Cat#A11039, RRID: AB_2534096; AF555, Cat#A31572, RRID: AB_162543; AF555, Cat#A21428, RRID: AB_2535849; AF647, Cat# A21235, RRID: AB_2535804) or CF® (633, Sigma-Aldrich, Cat#SAB4600128) dyes and DAPI for two hours at RT in the dark. The slides were mounted with Prolong gold (Invitrogen, California, USA, Cat# P36930) and imaged with Axioscan or Tissue FAXS Plus.

For immunohistochemistry (IHC) staining, the sections were first deparaffinized, rehydrated, and antigen retrieved. Then they were treated with 3% H_2_O_2_ (v/v) for 10 min to quench the endogenous peroxidase before blocking and primary antibody incubation. On the second day, the sections were incubated with HRP-conjugated secondary antibodies for two hours at RT. Then the sections were treated with Streptavidin HRP for 10 min and visualized by DAB substrate application (Abcam, Waltham, USA, Cat#ab64238). The cell nuclei were counterstained with Hematoxylin, then dehydrated and mounted as the hematoxylin and eosin (H&E) staining. For BrdU staining, the sections were treated with 2 N HCl for 5 min at 37 °C before blocking. The slides were imaged with Axioscan or Tissue FAXS Plus.

Primary antibody dilutions: Ki67 (Cell Signaling Technology, Cat#9129, RRID: AB_823664), 1: 400 for IF, 1:1000 for IHC; GFP (Abcam, Cat#ab13970, RRID: AB_300798), 1: 500 for IF; GFAP (Cell Signaling Technology, Cat#3670, RRID: AB_561049), 1: 1000 for IHC; BrdU (BD Biosciences, New Jersey, USA, Cat#347580, RRID: AB_10015219), 1: 500 for IHC; Nestin (Cell Signaling Technology, Cat#33475, RRID: AB_10015219), 1: 200 for IF, 1:1000 for IHC; SOX2 (Abcam, Cat#ab97959, RRID: AB_2341193), IF 1: 500, IHC 1:1000; Tubulin B3 (TUJ1) (Biolegend, California, USA, Cat#801202, RRID: AB_10063408), IF 1: 1000. FABP7 (BLBP) (Abcam, Cat#32423, RRID: AB_880078), IF 1:200; SOX9 (Abcam, Cat#ab185966, RRID: AB_2728660), IF 1:300; Doublecortin (DCX) (Santa cruz, Cat#sc-291390), IF 1:100.

### Cultivation of mouse glioma stem cells

The *Pten/Trp53* KO mouse glioma stem cells (BTSC, mGB2) were described previously^47^. The cells were cultured in N2 medium containing 97% DMEM/F12 (v/v), 1% Pen/Strep (Sigma-Aldrich, Cat#V900929), 1% GlutaMAX (v/v), 1% N2 (v/v), 20 ng/ml bFGF, and 20 ng/ml EGF (Invitrogen, Cat#PHG0311). Cells were split every 3 days.

### Animal experiments

Female NOD/SCID and C57BL/6 N mice were purchased from Shanghai Jihui Laboratory Animal Care Co.,Ltd (Shanghai, China) and housed in the Animal Facility at the National Facility for Protein Science in Shanghai. All mouse experiments were conducted under Shanghai Institutional Animal Care and Use Committee (IACUC) guidelines and an approved IACUC protocol of ShanghaiTech University (#20201208001) according to all relevant ethical regulations for animal testing and research.

### Mouse orthotopic xenograft

Single cells dissociated from the 45-day-old organoids induced from Luc2+ iPSCs (WT, PT, PTCC, PTN) were orthotopically injected into the right striatum of 4- to 5-week-old female NOD/SCID mice (WT, *N* = 11; PT, *N* = 9; PTCC, *N* = 10; PTN, *N* = 9). Briefly, organoids were cut into small pieces with scalpels and digested with the Neural dissociation kit (P) (Miltenyi, Bergisch Gladbach, Germany, Cat#130-092-628) following the manufacturer’s protocol. *Pten/Trp53* KO mouse BTSCs single cells digested with Accutase and injected into the right striatum of 4- to 5-week-old female C57/BL6N mice. After the dissociation, for both injections, 5 × 10^5^ cells were resuspended in 2 μL HBSS (Gibco, Cat# 14170088) and stored on ice. The mice were anesthetized with 0.15‰ avertin solution (1.2% avertin solution consists of 25 g of tribromoethanol (Sigma-Aldrich, Cat#T48402), and 15.5 ml of 2-Methyl-2-butanol (Sigma-Aldrich, Cat#152463) which dissolved in 0.9% saline), the cells were injected at the position 2 mm to the right lateral bregma and 3 mm deep with a flow of 0.2 µL/min utilizing a 10 µL precision micro syringe (World Precision Instruments, Florida, USA) with a 34-gauge needle. Mice were checked daily for signs of distress, including continuous weight loss or neurological disorders (such as hydrocephalus or impaired motor skills), and sacrificed with CO_2_ as soon as they showed related symptoms. The brains were collected for histological analysis.

### Single-cell RNA sequencing and data analysis

The single-cell RNA sequencing libraries were generated from 1- and 4-month-old whole organoid dissociations using Chromium Single Cell 3′ Kit v3.1 (10x Genomics, California, USA). One-month-old organoids (three organoids each group) were treated with Cell Recovery Solution (Corning, Cat#354253) for 20 min at 4 °C to remove the surrounding Matrigel, and 4-month-old organoids (three organoids each group) were cut into four pieces and washed with DPBS (Gioco, Cat# 14190144) for three times to remove dead cells from the inner core before cell dissociation with the Neural dissociation kit (P). After the dissociation, the single cell suspensions were filtered with a 100 μm cell strainer (Gibco) followed by 70 μm, and 40 μm Flowmi® cell strainers (Fisher Scientific, Cat#BAH136800070-50EA, Cat# BAH136800040-50EA). An equal number of cells from three separately digested organoids for each group were pooled and loaded onto the 10X Genomics microfluidics chip. The libraries were prepared according to the manufacturer’s protocols and sequenced using the NovaSeq 6000 Paired-End S1 kit (Illumina, California, USA) by the NGS Core Facility of the German Cancer Research Center (DKFZ).

Raw RNA-seq reads were aligned to human genome hg19 (Ensembl v75) with Cell Ranger (v3.1.0)^61^ with non-default parameter “—expect-cells=10000”. Data from WT, PT, PTCC, and PTN organoids at 1- and 4-month-old were aligned separately. Raw reads in each condition were analyzed with Seurat (v3.1.5)^62^. Briefly, cells with the number of features in the quantile range of 5% and 95% in populations, as well as with less than 10% of reads aligned to mitochondrial genes, were used for downstream analysis. We used 75 principal components for dimension reduction, cluster identification, and low-dimension projections. To perform RNA velocity analysis, the splicing information of cells was calculated for each organoid separately with velocyto (v.0.17.17)^63^. We generated the 1- and 4-month data by concatenate results across conditions. Looms were converted to h5ad files integrating cell annations and UMAP/t-SNE embedding. RNA velocity was estimated with the stochastic model with generated h5ad files as input to scvelo (v0.2.2)^64^. The tumor cell state was annotated by mapping the cluster gene signature to the reference cell state signatures^25^ with Fgsea R package^65^, the signature with the smallest *P* value was chosen as annotation, in the case when the *P* values were the same, the enrichment score and the gene expression was evaluated to determine the cluster annotation, the cluster remained unmapped if there was no significantly enrichment cell state. The tumor cell meta-module score and the annotation with normal cerebral organoid cell types were performed with the scallop R package^9^.

### DNA methylation array and data analysis

For the GFP+ iPSC cell line derived organoids, we have four samples each for 3-month-old LEGOs, three samples each for other groups. For the other iPSC cell line, we have three organoids from each group of 2-month-old organoids. The organoids were cut into four pieces and washed with DPBS 3 times to remove the dead cells. For both iPSC cell line, three samples each containing around 2 million cells were used for DNA extraction. The DNA from each genotype was extracted with Dneasy Blood & Tissue Kit (Qiagen, Hilden, Germany, Cat# 69504) following the manufacturer’s protocol. DNA methylation array analyses were then performed with Infinium Methylation EPIC BeadChip Kit (Illumina) or Infinium HumanMethylation450 BeadChip kit (Illumina) according to the manufacturer’s instructions by the microarray unit of the DKFZ Genomics and Proteomics Core Facility.

The DNA methylation EPIC array data were processed with the CHAMP R package^66^ following the recommended pipeline. A total of 740031 probes were kept for analysis after filtering and normalization. The PCA plot was drawn with the Factoextra R package. Differentially methylated probes (DMP) were identified for PT vs. WT, PTCC vs. WT, and PTN vs. WT at different time points. The differential methylation level of over-methylated and under-methylated probes was calculated by dividing the number of DMP by the total number of probes. The mean delta beta value of all the DMPs localized on specific gene features was used to rank the genes for gene set enrichment analysis (GSEA)^67^ by the Fgsea R package^65^. Methylation clustering was performed based on previously identified methylation classification probes^26^. The MGMT methylation level was calculated by the MGMT-STP27 logistic regression model using the M values of two probes (cg12434587 and cg12981137)^68^, and the M values were calculated by log transformation of the beta values (M = log_2_(beta/(1-beta))^69^. The gene ontology analysis of the stable probes was performed with the clusterProfiler R package^70^. The DNA methylation 450 K array data was analyzed with the CHAMP R package^66^, and The PCA plot was drawn with the Factoextra R package.

### Metabolome and lipidome sample processing and data analysis

#### Sample collection

We performed metabolome and lipidome profiling on 1- and 4-month-old organoids (three organoids each sample, five samples in each group) and corresponding culture medium (200 µL medium each sample, five samples in each group). Six organoids were transferred to each 6-well-plate well containing 3 mL culture medium and conditioned for two days at 37 °C with 5% CO_2_ on orbital shakers. Blank medium control was prepared by incubating fresh medium under the same condition without organoids. Three organoids were quickly washed with 154 mM ammonium acetate on ice and collected as one sample, and 300 μL medium was collected from each well. All the samples were snap frozen in liquid nitrogen and stored at −80 °C before extraction.

### Organoid extraction (water-soluble metabolites and lipids)

The organoid samples were homogenized with Mixer Mill (Retsch, Haan, Germany) and ceramic beads at maximum frequency for two to four minutes in pre-cooled racks after adding ice-cold methanol/H_2_O (4:1, v/v, 500 µL per 40 mg tissue) with internal standards (4 µM lamivudine, 4 µM D4-glutaric acid, 4 µM D8-phenylalanine, and 16 µl Splash Lipidomix per 40 mg tissue). 500 µL of homogenate was then collected and extracted by applying 60 µL 0.2 M HCl, 200 µL chloroform, 200 µL chloroform, and 200 µL H_2_O consecutively with vortex. The extracts were spun down at 16000 g for ten minutes, and the upper phase (water-soluble metabolites) was evaporated for 30 min at 35 °C under nitrogen and dried in SpeedVac (Eppendorf, Hamburg, Germany) at 15 °C overnight. The lower phase (lipids) was evaporated to dryness at 45 °C under nitrogen. The interphase was used to determine the protein concentration with the BCA assay. Samples were stored at −80 °C.

### Culture medium extraction for water-soluble metabolites

The water-soluble metabolites in the culture medium were extracted with RP18 SPE columns (Merck, Darmstadt, Germany, Cat#102014). Briefly, 50 µL medium was mixed with 50 µL H_2_O and 400 µL methanol/acetonitrile (5/3, v/v) containing internal standards (4 µM D4-glutaric acid, 4 µM D8-phenylalanine), vortexed and ultrasound for three minutes. The supernatants were then filtered through the RP18 SPE columns (activated by elution of 1 mL acetonitrile and equilibrated by elution 1 mL methanol/acetonitrile/H_2_O (5/3/2, v/v/v) before usage) after centrifugation (5 min, 16,000 × *g*, 4 °C). The eluents were collected and mixed with 400 µL of methanol/acetonitrile/H_2_O (5/3/2, v/v/v). The mixtures were vortexed, ultrasound, centrifuged, and filtered as before and the eluent was collected and evaporated in SpeedVac overnight at 15 °C. Samples were stored at −80 °C.

### Culture medium extraction for lipids

The lipids in the culture medium were extracted with methanol and chloroform. Briefly, 200 µL medium sample was mixed with 800 µL methanol containing internal standards (6 µL Splash Lipidomix). 120 µL 0.2 M HCl, 400 µL chloroform, 400 µL chloroform, and 400 µL H_2_O were added to the mix consecutively and vortexed. The lower phase of the spun-down samples was collected with a 200 µL micro syringe (Hamilton, Reno, USA) and evaporated to dryness at 45 °C under nitrogen. Samples were stored at −80 °C.

### LC-MS analysis of water-soluble metabolites

Water-soluble metabolites from organoid and culture medium samples were dissolved in 200 µl 5 mM ammonium acetate (in 75% acetonitrile (v/v)) before loading to LC/MS. LC-MS analysis was performed on an Ultimate 3000 HPLC system (Thermo Fisher Scientific) coupled with a Q Exactive Plus MS (Thermo Fisher Scientific) in both ESI positive and negative mode. The analytical gradients were carried out using an Accucore 150-Amide-HILIC column (2.6 µm, 2.1 mm × 100 mm, Thermo Fisher Scientific) with solvent A (5 mM ammonium acetate in 5% acetonitrile) and solvent B (5 mM ammonium acetate in 95% acetonitrile). 3 µl sample was applied to the Amide- HILIC column at 30 °C, and the analytical gradient lasted 20 min. During this time, 98% of solvent B was applied for one minute, followed by a linear decrease to 40% within five minutes and maintained for 13 min before returning to 98% in 1 min and appended with a 5-min equilibration step. The flow rate was maintained at 350 µL/min. The eluents were analyzed with MS in ESI positive/negative mode with ddMS2. The full scan at 70k resolution (69-1000 m/z scan range, 1e6 AGC-Target, 50 ms maximum Injection Time (maxIT)) was followed by a ddMS2 at 17.5k resolution (1e5 AGC target, 50 ms maxIT, 1 loop count, 0.1 s to 10 s apex trigger, 2e3 minimum AGC target, 20 s dynamic exclusion). The HESI source parameters were set as 30 sheath gas flow rate, 10 auxiliary gas flow rate, 0 sweep gas flow rate, spray voltage: 3.6 kV in positive mode, 2.5 kV in negative mode, 320 °C capillary temperature, and the heater temperature of auxiliary gas was 120 °C. The annotation of the metabolites was performed using the EI-Maven software (Elucidata, https://www.elucidata.io/el-maven) with an offset of ± 15ppm.

### LC-MS/MS analysis of lipids

The lipids from the organoid and culture medium samples were dissolved in 100 µl of isopropylalcohol (iPrOH) before loading. The analytical gradients were carried out using an Accucore C8 column (2.6 µm, 2.1 mm × 50 mm, Thermo Fisher Scientific) with solvent A (acetonitrile/H_2_O/formic acid (10/89.9/0.1, v/v/v)) and solvent B (acetonitrile/iPrOH/H_2_O/formic acid (45/45/9.9/0.1, v/v/v/v)). 3 µl sample was applied to the C8 column at 40 °C, and the analytical gradient lasted for 35 min. During this time, 20% of solvent B was applied for two minutes, followed by a linear increase to 99.5% within 5 min and maintained for 27 min before returning to 20% in 1 min and appended with a 5-min equilibration step. The flow rate was maintained at 350 µL/min. The full scan and ddMS2 parameters were the same as the analysis of the water-soluble metabolites, except the scan range were adjusted to 200-1600 m/z. The HESI source parameters were also adapted with a 3-sweep gas flow rate and a 3.2 kV spray voltage in positive and 3.0 kV in negative mode. Peaks corresponding to the calculated lipid masses (±5 ppm) were integrated using El-Maven software.

### Metabolome and lipidome data analysis

Two of the 4-month-old organoid samples (one in the PT group and one in the PTN group) were removed from downstream analysis due to the low signal intensity of the internal standards. For organoid sample normalization, the intensity of each target was normalized to respective internal standards (positive/negative standards for metabolites, lipid class standards for lipids) and the sample protein concentration. The intensities of medium samples were subtracted by the median of the blank medium before normalizing to internal standards and protein concentrations to visualize the changes driven by organoid metabolism. The metabolomics data was further normalized by variance stabilization normalization (VSN) with the VSN R package^71^ and significant pathways in the Small Molecule Pathway Database (SMPDB) were identified by the quantitative enrichment analysis with MetaboAnalystR^72^. The lipidomics data were further normalized by quantile normalization with the Limma R package^73^, and the enrichment was calculated by comparing the structural similarities with ChemRich^74^.

### Proteome and phospho-proteome sample processing and data analysis

#### Sample preparation

The proteomics and phospho-proteomics samples (five samples in each group, three organoids each sample) were prepared according to a previously published protocol with adaptations^75^. Briefly, cell pellets were resuspended with lysis buffer (100 mM Tris-HCl pH 8.5, 7 M Urea, 1% Triton, 10 U/mL Dnase I (1 mM magnesium chloride, 1% benzonase, 1 mM sodium orthovanadate, phosphoSTOP phosphatases inhibitors, complete mini EDTA free protease inhibitors) and lysed by sonication. Cell debris was removed by 1.5 hours of 17000 g centrifugation at 4 °C. 1% benzonase was added to the supernatant, followed by incubation at RT for two hours. Protein concentration was determined by the Bradford assay. Proteins were precipitated using chloroform/methanol^76^, and the pellets were resuspended (8 M Urea, 100 mM NaCl, 50 mM triethylammonium bicarbonate (TEAB), pH 8.5) and reduced in 10 mM dithiothreitol (DTT) for one hour at 27 °C, then alkylated by 30 mM Iodoacetamide for 30 min at RT in the dark and the reaction was quenched by adding additional 10 mM DTT. Samples were subsequently digested by Lys-C at an enzyme: protein ratio of 1:100 for four hours at 30 °C, diluted with 50 mM TEAB to a resulting Urea concentration of 1.6 M, and further digested with Trypsin overnight at 37 °C in an enzyme: protein ratio of 1:50. Digestion was stopped by acidification using 0.02% trifluoroacetic acid (TFA, v/v). Digested peptides were desalted using C18 SepPack Cartridges (Waters) and resuspended in 0.07% TFA (v/v) in 30% acetonitrile (v/v) and fractionated by on-column FE^3+^- Immobilized Metal Ion Affinity Chromatography (IMAC) enrichment on an Ultimate 3000 LC system using the method described previously^77^. The two resulting fractions per sample, containing either unphosphorylated or phosphorylated peptides, were desalted by StageTips^78^. Before LC-MS/MS analysis, the dry peptides were resolved in 50 mM citric acid and 0.1% TFA.

### LC-MS/MS analysis of proteomics

LC-MS/MS analysis was carried out on an Ultimate 3000 UPLC system directly connected to an Orbitrap Exploris 480 mass spectrometer (Thermo Fisher Scientific). Peptides were online desalted on a trapping cartridge (Acclaim PepMap300 C18, 5 µm, 300 Å wide pore, Thermo Fisher Scientific) for three minutes using 30 µL/min flow of 0.05% TFA in water. The analytical multistep gradient was carried out using a nanoEase MZ Peptide analytical column (300 Å, 1.7 µm, 75 µm x 200 mm, Waters) using solvent A (0.1% formic acid in water) and solvent B (0.1% formic acid in acetonitrile). A total of 150 min of LC-MS/MS analysis time was used per sample. The analytical step of the gradient was 134 min, during this time, the concentration of B was linearly ramped from 4% to 30% (2% to 28%, for phospho-peptides), followed by a quick ramp to 78%, and after two minutes the concentration of B was lowered to 4% (2% for phospho-peptides) and a 10 min equilibration step appended. Eluting peptides were analyzed with the mass spectrometer using data-dependent acquisition (DDA) mode. A full scan at 120k resolution (380-1400 m/z, 300% AGC target, 45 ms maxIT) was followed by up to 2 seconds of MS/MS scans. Peptide features were isolated with a window of 1.4 m/z (1.2 m/z for phospho-peptides) and fragmented using 26% NCE (28% NCE for phospho-peptides). Fragment spectra were recorded at 15k resolution (100% AGC target, 22 ms maxIT; 200% AGC target, 54 ms maxIT for phospho-peptides). Unassigned and singly charged eluting features were excluded from fragmentation, and dynamic exclusion was set to 35 seconds (10 seconds for phospho-peptides).

### Target identification and data analysis

Data analysis was carried out by MaxQuant^79^ (version 1.6.14.0) using an organism-specific database extracted from Uniprot.org under default settings. Identification FDR cutoffs were 0.01 on the peptide level and 0.01 on the protein level. For the phospho enriched fraction, PTM was set to True and Phospho (STY) was added as variable modification. The full proteome samples were given a separate parameter group with the default variable modifications. The match between runs (MBR) option was enabled to transfer peptide identifications across RAW files based on accurate retention time and mass-to-charge ratio. The fractions were set in a condition that MBR was only performed within phospho enriched and full proteome and within each condition. The full proteome quantification was done based on the MaxLFQ algorithm^80^. A minimum of two quantified peptides per protein was required for protein quantification. LFQ, and phosphosite intensities were filtered for target groups with a non-zero intensity in 70% of the samples of at least one of the conditions and normalized via VSN^71^. For missing values with no complete absence in one condition, the R package missForest^81^ was used for imputation. The missing values that were completely absent in one condition were imputed with random values drawn from a downshifted (2.2 standard deviations) and narrowed (0.3 standard deviations) intensity distribution of the individual samples^82^. The significance for each target was then calculated with Student’s t-test and adjusted with Benjamini–Hochberg method.

The protein abundances of GB subtype signature genes^42^ were plotted, and the enrichment P value was calculated with the “ssgsea.GBM. classification” R package^42^. Enrichment analysis of the full proteome was carried out with the GSEA software (NIH Broad Institute, version 4.2.3) on Hallmark, KEGG, Reactome, and GO biological process gene sets. Potential druggable targets were identified by mapping the significantly differentially expressed proteins (*P* adjusted < 0.05 and foldchange (FC) > 1) to a drug-gene interaction database DGIdb^43^, and the combinations with an interaction group score more than 5 were kept. The upregulated phospho-sites (FC > 1) were mapped to a kinase/substrate interaction database^44^ to identify upstream kinases, and the *P* value for each kinase was calculated by Kinase Enrichment Analysis^45^. The interactions were visualized by Cytoscape^83^ (version 3.9.1), and the largest subnetwork was shown in the figure.

### RNA sequencing and data analysis

We performed bulk-RNA sequencing on 2-month-old organoids derived from both iPSCs (three organoids each sample, three samples in each group), the RNA was extracted with Qiagen Rneasy kit (Cat#74004). The RNA was extracted and purified according to the protocol, and the library preparation and sequencing with Illumina NovaSeq were performed by our in-house facility. One WT sample failed sequencing and was excluded from the following analysis.

Raw reads were aligned to GRCh38 with STAR (2.4.1)^84^ using default parameters, the aligned reads were quantified by the feature Counts in Rsubread package^85^ and TPM were then calculated and used for following PCA analysis. Enrichment analysis was carried out with the GSEA software (NIH Broad Institute, version 4.2.3)^67^ on Hallmark, KEGG, Reactome, and GO biological process gene sets by group comparison.

### Organoid drug screen

Drug screens on several selected kinase inhibitors (Sellekchem), TMZ (Sigma), one library containing FDA-approved drugs that can penetrate through blood–brain barrier (269 drugs from TargetMol, Massachusetts, USA), and one library containing drugs targeting the possible targets identified in omics analysis (58 drugs from MedchemExpress, New Jersey, USA). A list of drug information can be found in Supplementary Table 6.

For drug screening, the organoids generated from Luc2+ iPSCs were cultured with the culture medium containing kinase inhibitors (5 days, 10 µM, applied daily), TMZ (6 days, 100 µM, applied every other day), drug libraries (6 days, 10 µM, applied every other day) or DMSO vehicle on the orbital shakers at 37 °C with 5% CO_2_. BLI was performed before drug administration to one (for daily administration) or two (for every other day administration) days after the last dose. For BLI, the organoids were incubated with 150 μg/mL D-luciferin in a 37 °C incubator supplied with 5% CO_2_ on the orbital shakers for 15 min and then imaged with IVIS or Quick View 3000 (Bio Real, Salzburg, Austria). To assess the treatment effects, the BLI signals were normalized to the DMSO control measured on the same day, then compared to before treatment. Drugs with a *P* value less than 0.05 and a signal drop of more than 50% were considered effective. In addition, 100 μM BrdU (Sigma-Aldrich) was applied to the culture medium after the final imaging and cultured for 2 hours, and the samples were collected and stained as described above.

### Organoid IC50 analysis

For drug testing, WT, PT, PTCC, and PTN organoids were exposed to Aripiprazole, Osimertinib, and Lomitapide treatments over a 6-day period (applied every other day). The drug concentrations administered to PT, PTCC, and PTN organoids were 0 μM, 0.01 μM, 0.1 μM, 1 μM, 3 μM, 7.5 μM, 10 μM, and 100 μM. BLI signals were measured and analyzed as described in “Organoid drug screen” section. The IC50 of each drug was calculated using Graphpad Prism 8 (San Diego, CA, USA). Subsequently, the maximum IC50 concentrations of the drugs were administered to WT organoids, and BLI signals were measured and analyzed in the same manner.

### Mouse treatment

Lomitapide (MedchemExpress, Cat#HY-14667) was dissolved in DMSO and freshly diluted in a solution containing 40% PEG400 (Sigma-Aldrich, Cat#81172), 5% Tween-80 (Sigma-Aldrich, Cat#W291706), and 45% saline, resulting in a 5.2 mg/ml solution. DMSO was diluted using the same procedure. 2 μL volume of Lomitapide or DMSO was injected at a position 2 mm to the right of the lateral bregma and 3 mm deep, with a flow rate of 0.2 µL/min, using brain infusion cannulas (Yuyanbio, Shanghai, China, Cat#SL-4) over 5 consecutive days (DMSO, *N* = 9; Lomitapide, *N* = 9). Mice were monitored daily for signs of distress, including continuous weight loss or neurological disorders (such as hydrocephalus or impaired motor skills), and were euthanized with CO_2_ as soon as related symptoms appeared. The collected brains were subjected to histological analysis.

### *Mttp* knockout and assays on *Pten/Trp53* KO mouse BTSCs

Cas9 ribonucleoproteins (RNPs) were prepared immediately before experiments by incubating 16 μg Cas9 protein (Sino Biological, Beijing, China, Cat#40572-A08B) with 0.125 nM *Mttp* sgRNA (Genescript, Nanjing, China) at 37 °C for 15 min. *Pten/Trp53* KO mouse BTSCs were harvested using Accutase and then electroporated with Cas9 RNPs using the EN100 program on the 4D-Nucleofector® Core, X, Y Unit (Lonza, Basel, Switzerland, Cat#AAF-1003B). The electroporated cells were cultured in N2 medium for 48 hours after electroporation and then seeded as single cells per well in a 96-well plate for 10 days. The single-cell spheres were screened by Sanger sequencing (Biosune, Shanghai, China), and the results were analyzed using the TIDE web tool^57^. Two clones were selected and further validated by western blotting and Lomitapide treatment. For Lomitapide treatment, the cells were exposed to Lomitapide at 500 nM and 1000 nM for 48 hours, and then cell viabilities were assessed using CellTiter-Glo (Promega, Cat#G7572) following the manufacturer’s instructions. Luminescent signals were measured with an EnVision Plate Reader (PerkinElmer, Waltham, USA). gRNA sequences (5’ to 3’): *Mttp*, GGAAAACCGCAAGACAGCGT. Antibody dilutions: MTP (Santa Cruz, Cat#sc-515742), 1:1000; α-Tubulin (Sigma-Aldrich, Cat#T9026), 1:3000.

For the proliferation assay of *Mttp* WT and KO mouse BTSCs, the cells were dissociated using Accutase, and 5000 cells were plated in a single well of a 96-well plate, then cultured for 2 days in N2 medium. Cell viability in each well was assessed using CellTilter-Glo (Promega, Cat#G7572) following the manufacturer’s instructions. Luminescent signals were measured with an EnVision Plate Reader (PerkinElmer, Waltham, USA).

In the limiting dilution assay of *Mttp* WT and KO mouse BTSCs, 2, 5, 10, 15, and 20 cells were seeded into 20 wells of a 96-well plate each. After 2 weeks, the number of wells containing neurospheres was counted. The significance of the limiting dilution assay was analyzed using the ELDA online tool^86^.

### Quantification and statistical analysis

All the data analysis was performed with R (version 4.1.2), Graphpad Prism 8 and Microsoft Excel. The 2D areas of the organoids were measured with ImageJ (NIH) and the comparison was carried out with two-way ANOVA by normalizing the size of the organoids to match the average size of each group measured on day 5. For cell number quantification, positive cells were manually counted using the cell counter function in ImageJ. Group comparisons of Kaplan-Meier survival analysis of xenografted mice and mice treatment were calculated with Log-rank test. Students’ t-tests were applied when comparing variables between two groups (paired t-test for drug treatment and heteroskedastic for the rest). N numbers for each experiment can be found in the corresponding figure legend. All data values were presented as mean ± SEM and the *P* values are represented as follows: ****P < 0.0001, ***P < 0.001, **P < 0.01, *P < 0.05, and *P* > 0.05 is recognized as non-statistically significant (ns).

### Reporting summary

Further information on research design is available in the Nature Research Reporting Summary linked to this article.

### Supplementary information

Supplementary information

Reporting summary

Supplementary Table 2. Stable probe set-related to Fig. 3

Supplementary Table 3. Proteomics GSEA-related to Fig. 6

Supplementary Table 4. Proteomics drug targets-related to Fig. 6

Supplementary Table 5. Kinase enrichment analysis-related to Fig. 6

Supplementary Table 6. Drug screen-related to Fig. 7

## Data Availability

ScRNA-seq, DNA methylation array and RNA-Seq data have been deposited at GEO under accession codes GSE213835, GSE213554 and GSE247890. Metabolomics and lipidomics data are available at the NIH Common Fund’s National Metabolomics Data Repository (NMDR) website, the Metabolomics Workbench, (https://www.metabolomicsworkbench.org)^87^ where it has been assigned Study ID ST002284. The proteomics and phospho-proteomics data have been deposited to the ProteomeXchange Consortium via the iProX partner repository^88^ with the dataset identifier PXD036874. All data are publicly available as of the date of publication. Cell lines generated in this study will be made available with MTA upon request, further information and requests for resources and reagents should be directed to and will be fulfilled by the lead contact Hai-Kun Liu (L.Haikun@Dkfz.de).

## Code availability

This study didn’t generate original codes. Any additional information required to reanalyze the data reported in this paper is available from the lead contact upon request.
