# Supplementary material for: A multidimensional atlas of human glioblastoma-like organoids reveals highly coordinated molecular networks and effective drugs
Source: NPJ Precis Oncol. 2024 Jan 26;8:19. doi: 10.1038/s41698-024-00500-5 (PMC10811239; doi:10.1038/s41698-024-00500-5)
Supplement: Supplementary file 1 — Supplementary information [file 41698_2024_500_MOESM1_ESM.pdf]

Supplementary figures and tables

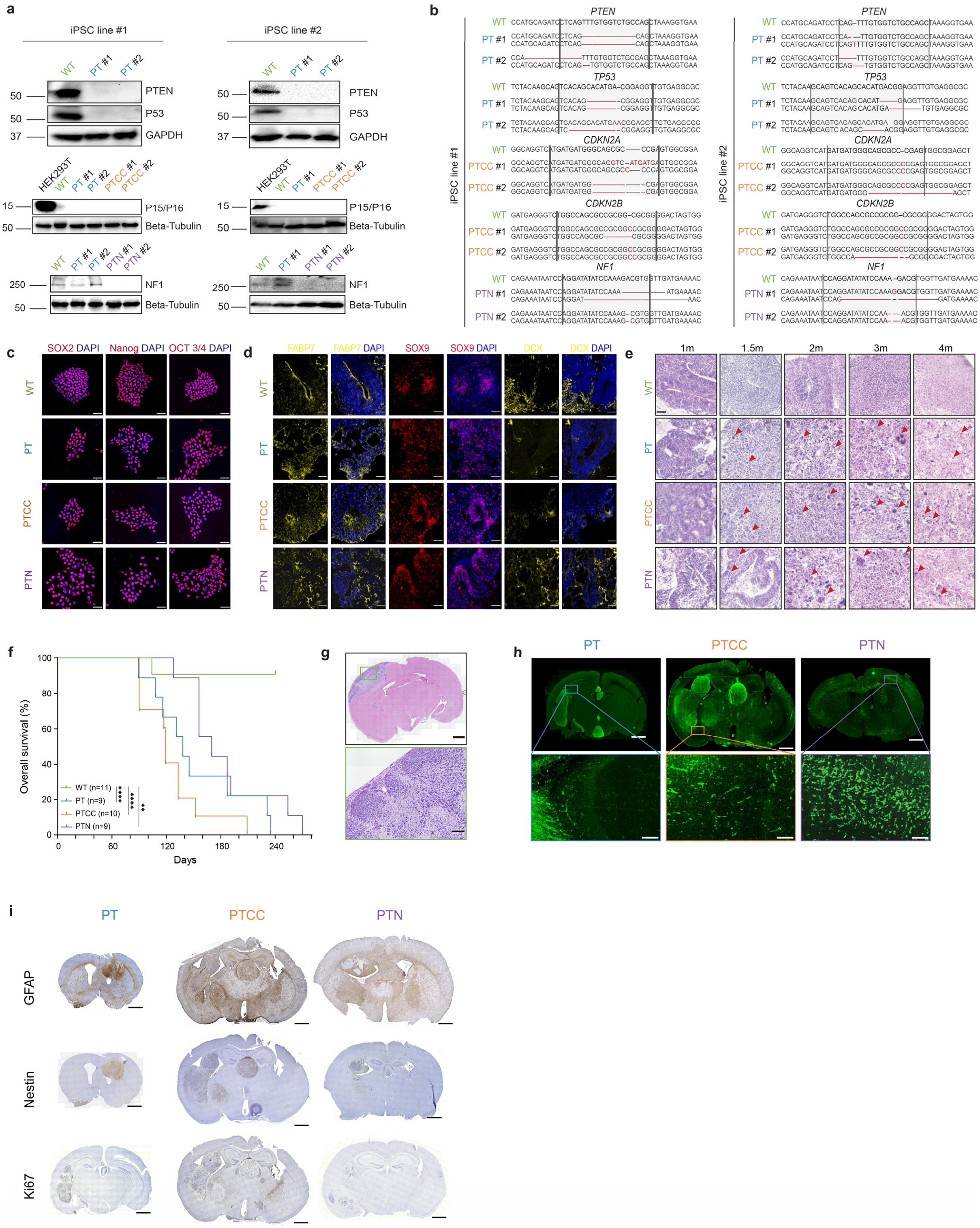

Supplementary Fig. 1. Related to Fig. 1

(a). Western blotting results of the knockout iPSC clones. HEK293T protein lysate serves as a positive control for P15/P16 detection. The experiments were each performed three times independently.

(b). Sanger sequencing results at the CRISPR/Cas9 gene-editing site of respective iPSC clones. gRNA sequences are highlighted with gray backgrounds, and mutations are highlighted in red.

(c). iPSC staining with pluripotent stem cell markers SOX2, Nanog and OCT 3/4. Scale bars 50 μm.

(d). Representative immunofluorescent staining images of 1-month-old organoids stained with FABP7, DCX and SOX9. Scale bars, 50 μm.

(e). Representative H&E staining images of the organoids at different ages. Red arrows indicate cells with atypical nuclei. Scale bars, 50 μm.
(f). The Kaplan-Meier survival analysis of WT organoids xenografted mice compared to LEGOs. n numbers are labeled in the figure. *P* values were calculated with Log-rank test.
(g). Representative H&E staining images of implanted WT organoid in mouse xenograft. Scale bar, 1000 μm for the overview, and 100 μm for insets.
(h). Representative GFP staining images showing the infiltrative growth pattern of mouse xenografts. 1000 μm for overview, and 100 μm for insets
(i). Representative IHC staining images with glioma-related markers. Scale bar, 1000 μm.
See also Supplementary Table 1.

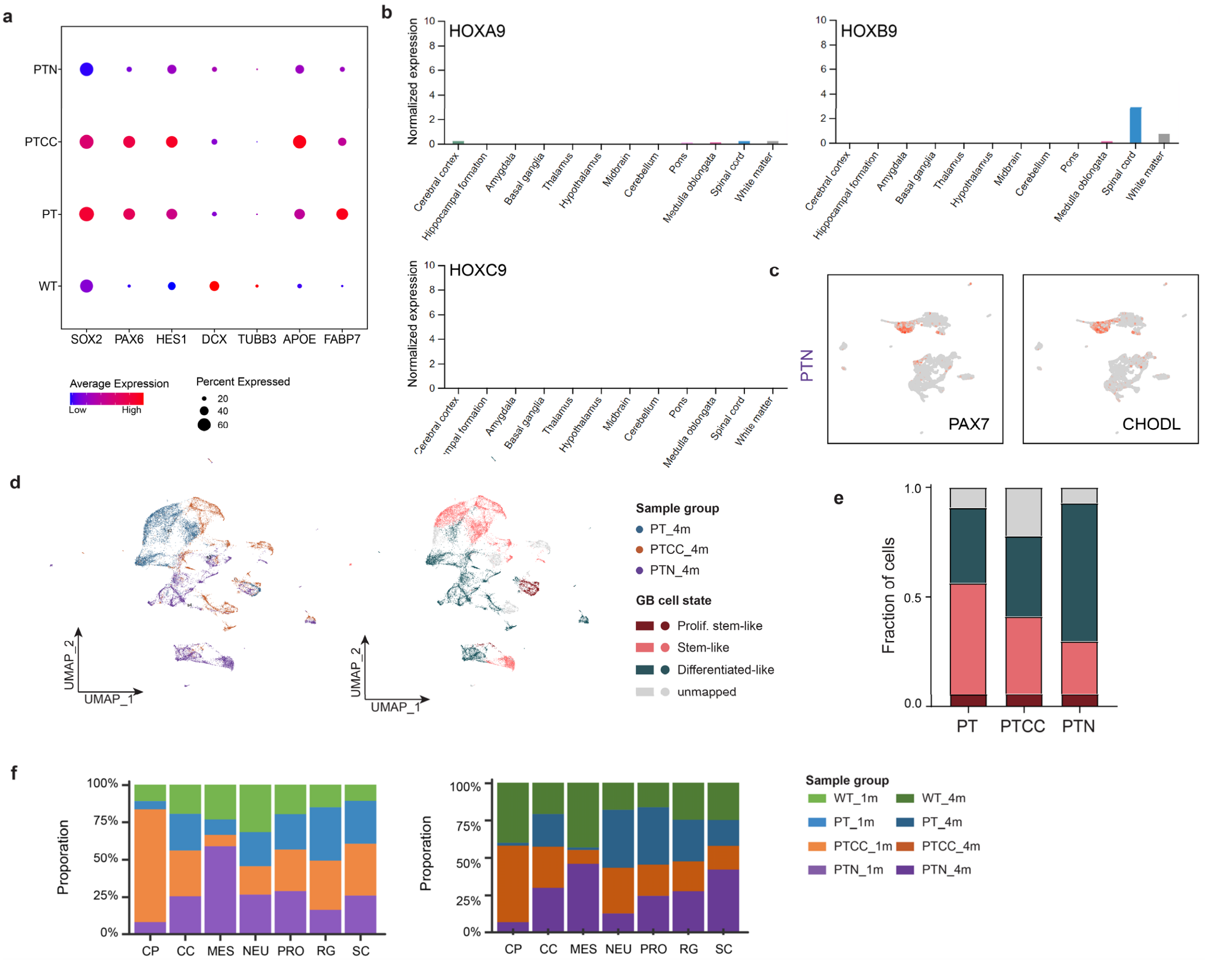

Supplementary Fig. 2. Related to Fig. 2

(a). Expression portion of lineage markers in different samples.

(b). Normalized RNA expression of HOX genes in different central nervous system regions from the Human Protein Atlas [1].

(c). UMAP gene expression plots of PAX7 and CHODL in four-month-old PTN organoids.

(d). UMAP for four-month-old LEGOs colored by sample (left) and GBM cell state (right) [2,3].

(e). Cell state proportion of four-month-old LEGOs.

(f). Distribution of genotypes among different cell types in one- and four-month-old organoids.

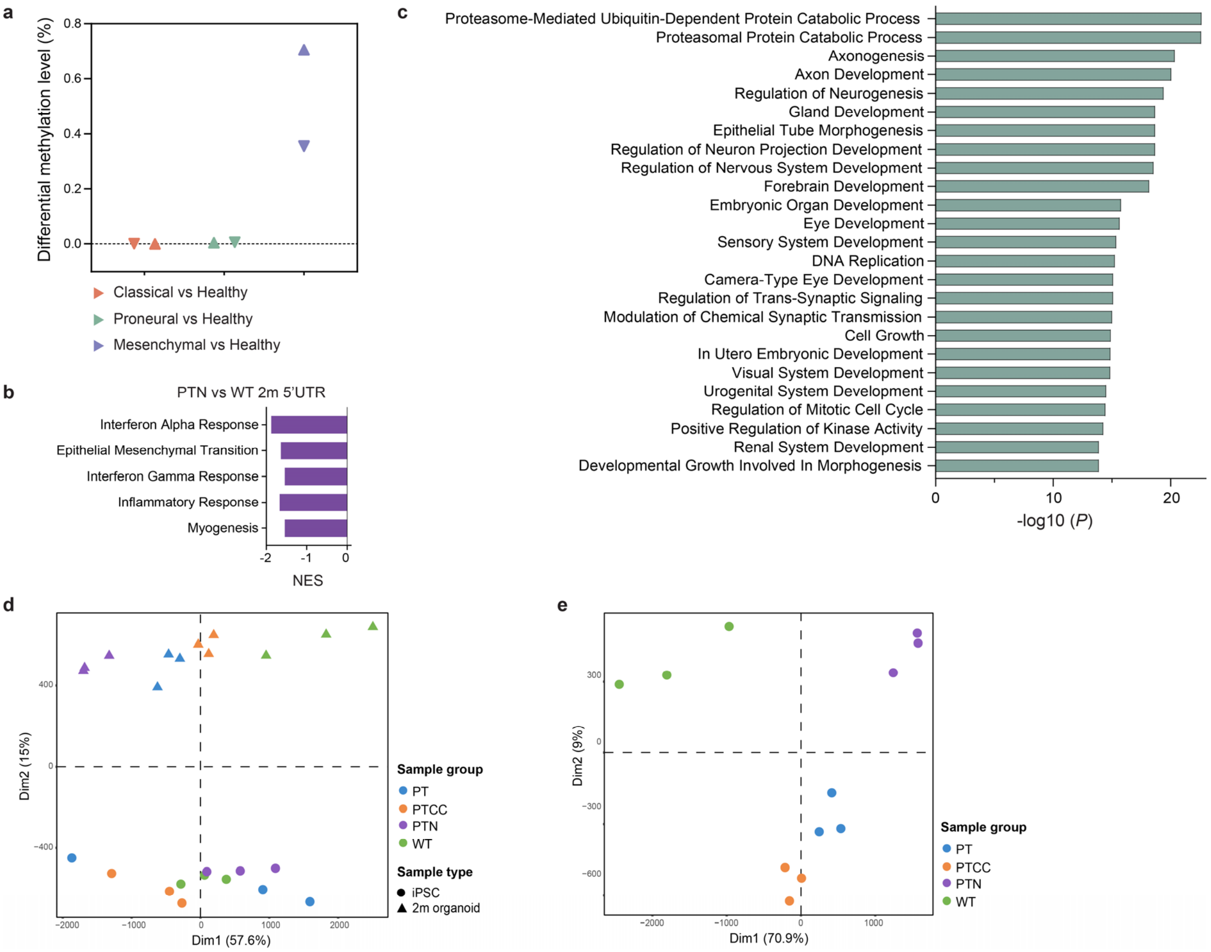

Supplementary Fig. 3. Related to Fig. 3.
(a). Differential methylation level of an external dataset [4] comparing different GBM transcription subtypes to the healthy control.
(b). GSEA hallmark enrichment of the DMPs located on 5'UTR in 2-month-old PTN organoids (adjusted *P* value < 0.05).
(c). The top 25 enriched gene ontology terms of the genes that the stable probes represent.
(d). PCA analysis comparing the DNA methylome of iPSCs to 2-month-old organoids.
(e). PCA analysis comparing the DNA methylome of 2-month-old organoids derived from another iPSC line.

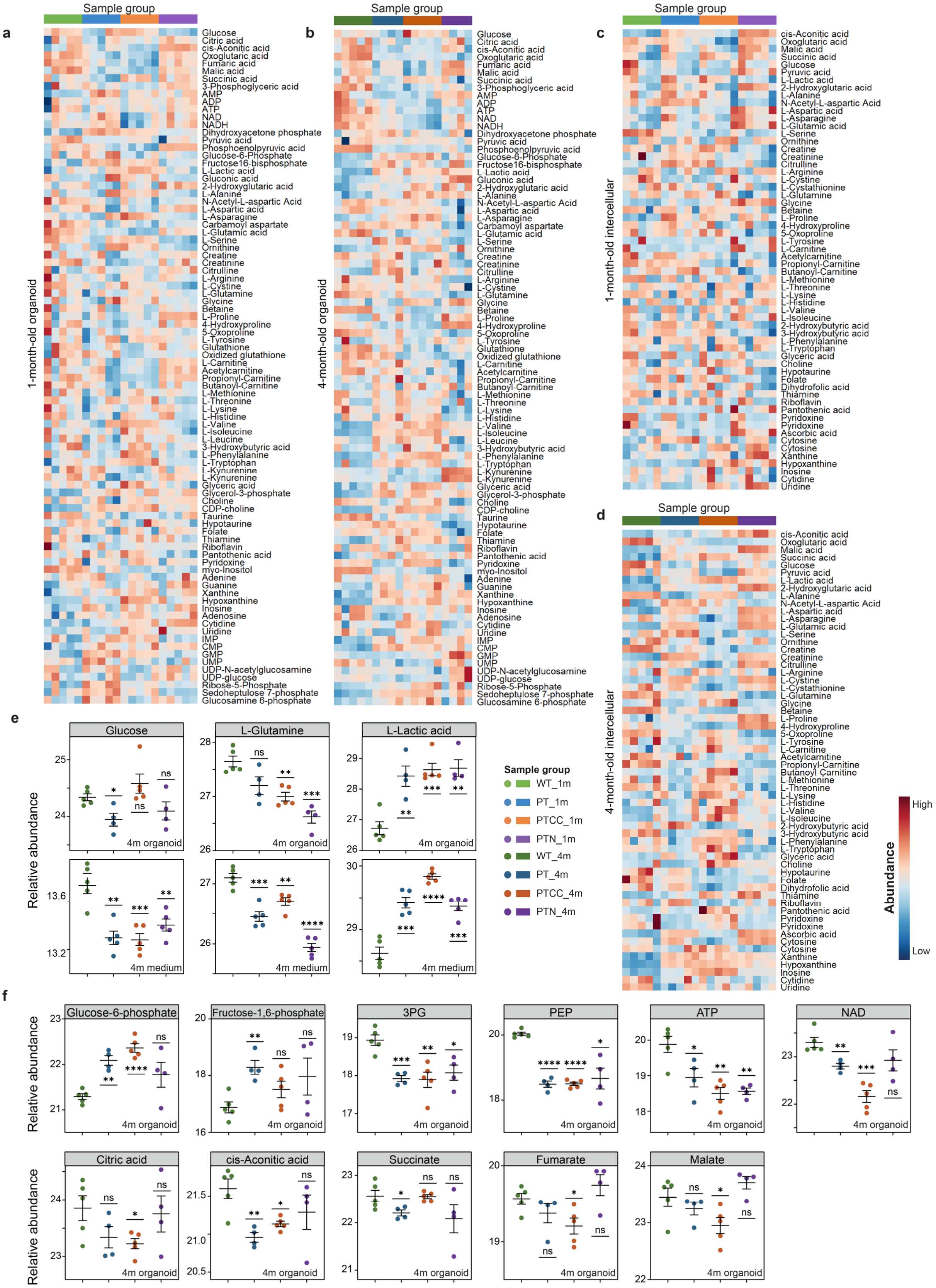

Supplementary Fig. 4. Related to Fig. 4

(a-d). Heatmap representation of metabolites in one-month-old organoids (a), four-month-old organoids (b), culture medium of one-month-old organoids (c) and culture medium of four-month-old organoids (d).

(e). The relative abundance of energy sources (Glucose and Glutamine) and glycolysis product (lactic acid) in four-month-old organoids and culture medium.

(f). The relative abundance of glycolysis and TCA cycle intermediates in four-month-old organoids. 3PG, 3-phosphoglyceric acid; PEP, phosphoenolpyruvic acid.

In e and f, the color of the dots indicates the sample groups, data are represented as mean ± SEM; N = 4 for four-month-old PT and PTN organoid samples, and N = 5 for the rest of the groups; statistical significances were calculated using Student's t-tests comparing respective mutant groups to WT; **** $P < 0.0001$, *** $P < 0.001$, ** $P < 0.01$, * $P < 0.05$, and ns, non-significant.

a

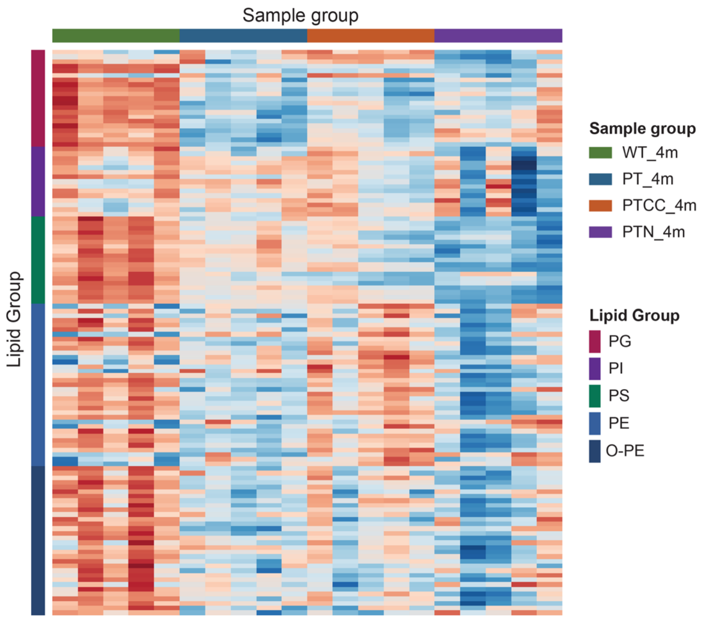

Supplementary Fig. 5. Related to Fig. 5
(a). Abundance heatmap of structure phospholipid species in four-month-old organoids.

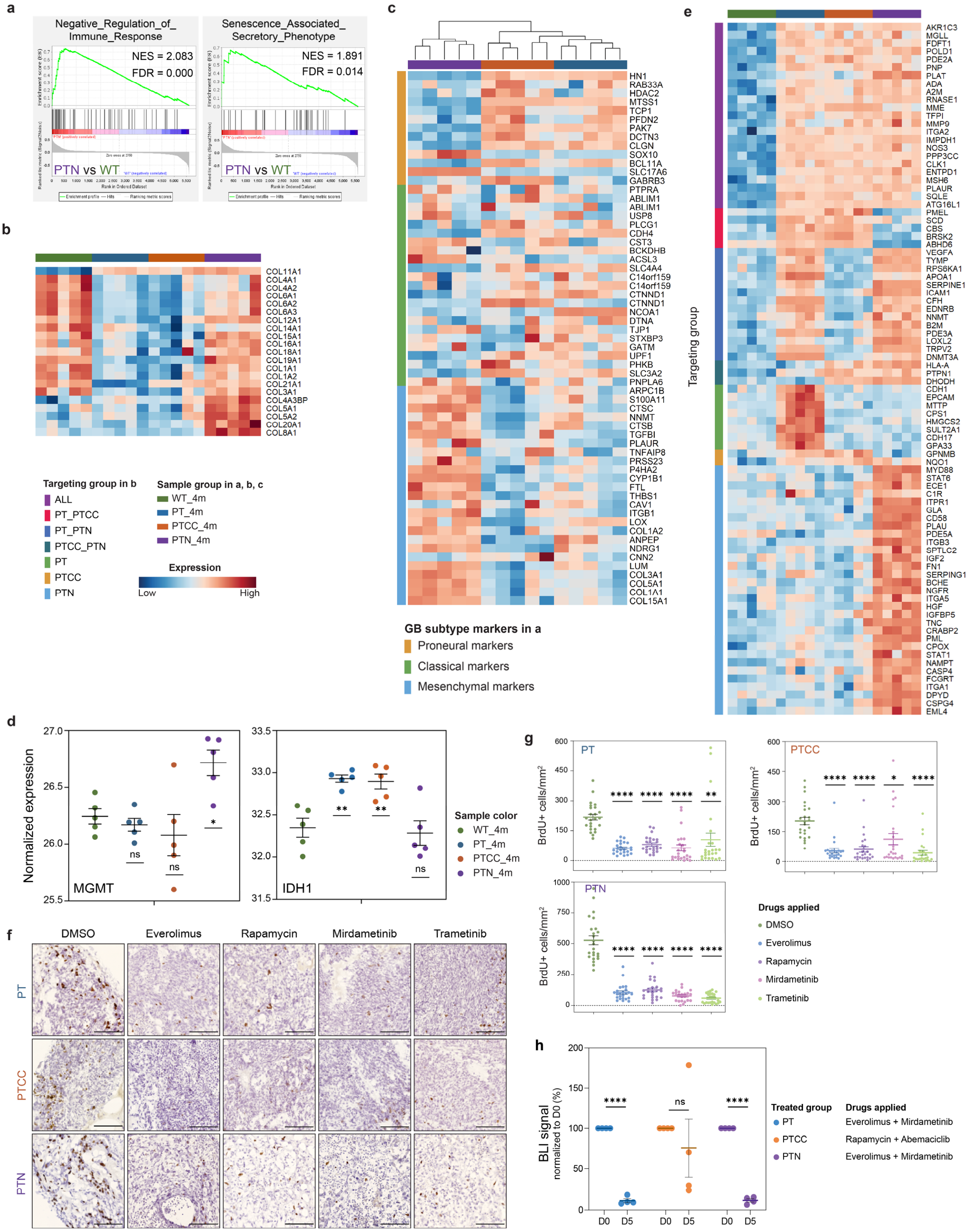

Supplementary Fig. 6. Related to Fig. 6

(a). GSEA enrichment plots for distinct enriched signatures in PTN.

(b). Heatmap of collagen protein abundance.

(c). Heatmap of protein levels relevant for GB transcriptomic subtype classification [5].

(d). Normalized expression of MGMT and IDH1 protein in different groups of organoids. N = 5 organoids for each group.

(e). Genotype specific drug targets shown in heatmap.

(f). Representative BrdU staining images of LEGOs after drug treatment. Scale bar, 100 μm.

(g). Quantification of BrdU+ cells after drug treatment. N = 24 sections for each group.

(h). Combination treatment in different LEGOs. N = 4 organoids for each group.

In d, g and h, the color of the dots indicates the group of organoids; data are represented as mean ± SEM; statistical significances were calculated with Student's t-test; **** P <0.0001, *** P < 0.001, ** P < 0.01, * P < 0.05, ns, non-significant.

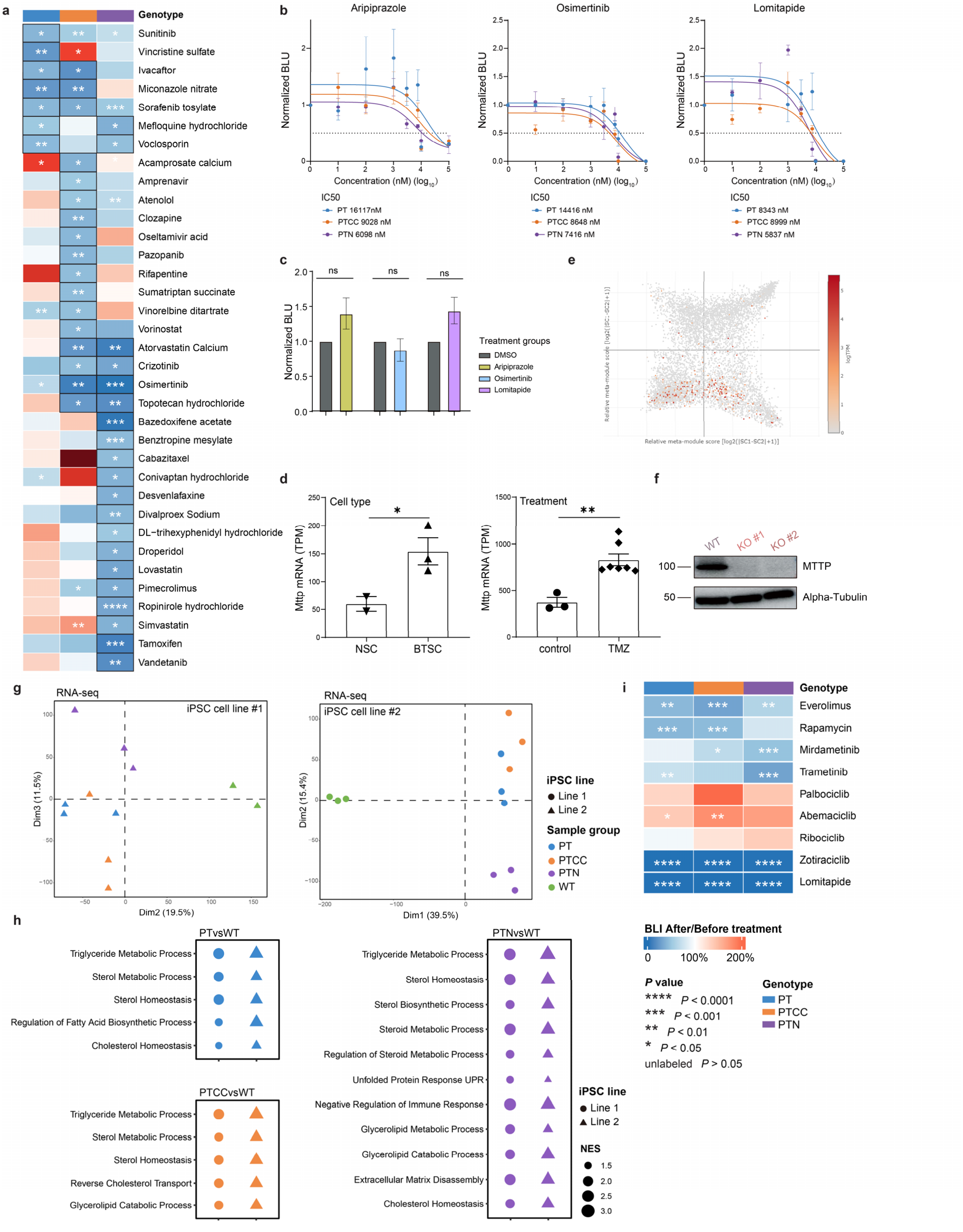

Supplementary Fig. 7. Related to Fig. 7

(a). Treatment outcome for drugs that are effective in at least one group. The black frames around the cells highlight the effective group. N = 3 for each group.

(b). IC50 analysis on the LEGOs, IC50 values were labeled at the bottom of each figure.

(c). Treatment of WT organoids with the maximum IC50 values derived from LEGOs. N = 4.

(d). Normalized Mttp expression from ribo-seq analysis of mouse BTSC and NSC (left) and RNA-seq analysis of mouse brain tumors treated with/without TMZ (right).

(e). MTTP expression from GBM patient single-cell RNA sequencing data.

(f). Western blot validation of *Mttp* knockout in *Pten/Trp53* KO mouse BTSCs.

(g). PCA applied to RNA sequencing results obtained from two-month-old organoids derived from two distinct iPSC lines.

(h). GSEA conducted on LEGOs in comparison to the WT across both iPSC lines.
(i). Outcomes of the treatment responses observed in the proof-of-principle drug tests. (N = 4).
In a and i, *P* values were calculated with paired Student's t-tests comparing signals measured after treatments with those measured before treatments; **** $P < 0.0001$, *** $P < 0.001$, ** $P < 0.01$, * $P < 0.05$. In b, c and e, data are represented as mean ± SEM.

Raw data images

Supplementary Fig. 1a

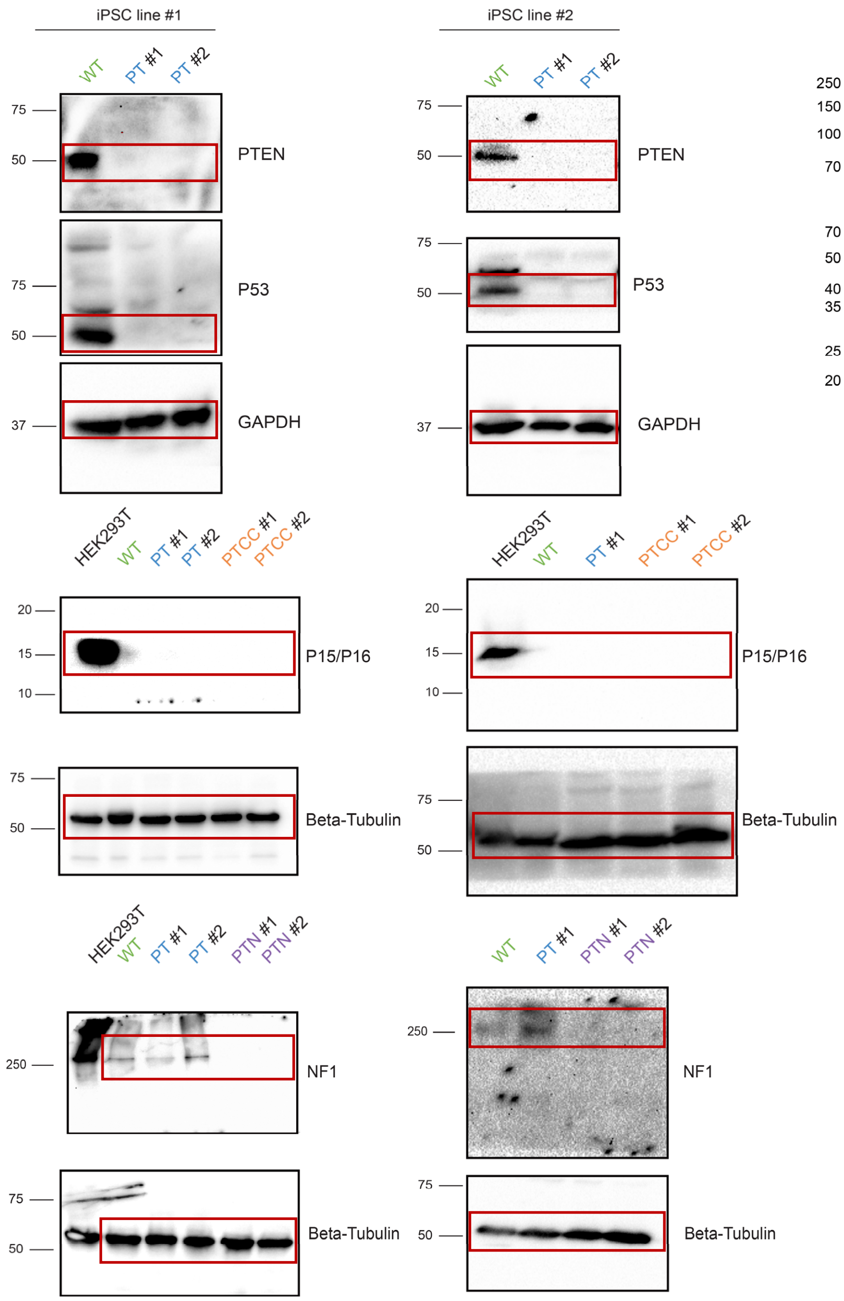

Supplementary Fig. 7f

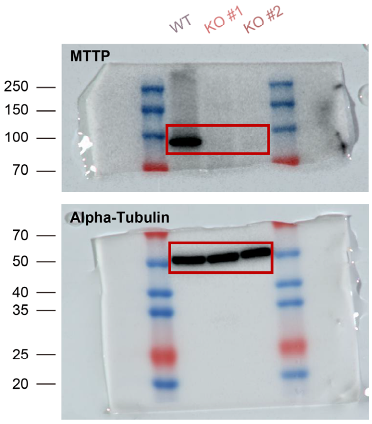

Supplementary Table 1. Xenograft mice observation-related to Fig. 1

Group	Days	Status	Observation	N number of each group
**WT**	104	1	Body weight unaffected; normal behavior	11
**WT**	240	0	Body weight unaffected; normal behavior	
**WT**	240	0	Body weight unaffected; normal behavior	
**WT**	240	0	Body weight unaffected; normal behavior	
**WT**	240	0	Body weight unaffected; normal behavior	
**WT**	240	0	Body weight unaffected; normal behavior	
**WT**	240	0	Body weight unaffected; normal behavior	
**WT**	240	0	Body weight unaffected; normal behavior	
**WT**	240	0	Body weight unaffected; normal behavior	
**WT**	240	0	Body weight unaffected; normal behavior	
**WT**	240	0	Body weight unaffected; normal behavior	
**PT**	89	1	Weight reduction >20%; hyperkinetic	9
**PT**	108	1	Weight reduction >20%; fur defects (decrease body care); lethargy	
**PT**	116	1	Weight reduction >20%; slight hydrocephalus	
**PT**	131	1	Weight reduction >20%	
**PT**	138	1	Weight reduction >20%; fur defects (decrease body care)	
**PT**	145	1	Weight reduction 5-10%; critically moribund condition; extremely labored respiration; profound loss of vitality	
**PT**	187	1	Weight reduction >20%	
**PT**	231	1	Weight reduction >10%; critically moribund condition; extremely labored respiration; profound loss of vitality	
**PT**	235	1	Weight reduction >20%	
**PTCC**	90	1	Weight reduction >20%	10
**PTCC**	90	1	Weight reduction >20%; fur defects (decrease body care)	
**PTCC**	90	1	Weight reduction >20%	
**PTCC**	117	1	Weight reduction >20%; critically moribund condition; extremely labored respiration; profound loss of vitality	
**PTCC**	119	1	Weight reduction >20%; medium hydrocephalus	
**PTCC**	119	1	Weight reduction >20%; fur defects (decrease body care)	
**PTCC**	133	1	Weight reduction >20%; pronounced hyperkinetic	
**PTCC**	133	1	Weight reduction >10%; critically moribund condition; extremely labored respiration; profound loss of vitality	
**PTCC**	152	1	Weight reduction >20%	
**PTCC**	209	1	Weight reduction >20%; slight hydrocephalus	
**PTN**	128	1	Weight reduction >20%	9
**PTN**	156	1	Weight reduction >10%; critically moribund condition; extremely labored respiration; profound loss of vitality	
**PTN**	156	1	Weight reduction >10%; profound loss of vitality; fur defects (decrease body care)	
**PTN**	156	1	Weight reduction >20%	
**PTN**	170	1	Weight reduction >20%; hyperkinetic	

**PTN**	187	1	Weight reduction >20%; critically moribund condition; extremely labored respiration; profound loss of vitality	
**PTN**	191	1	Weight reduction >20%; hyperkinetic	
**PTN**	254	1	Weight reduction >20%; uncoordinated	
**PTN**	270	1	Weight reduction >20%	

Supplementary Table 7. Mouse treatment observation-related to Fig. 7

Group	Days	Status	Observation	N number of each group
**DMSO**	50	1	Weight reduction >20%; fur defects (decrease body care); seizures; automatisms; cerebral hemorrhage	9
**DMSO**	52	1	Weight reduction >20%; fur defects (decrease body care); strong hydrocephalus	
**DMSO**	52	1	Weight reduction 5-10%; critically moribund condition; extremely labored respiration; profound loss of vitality	
**DMSO**	61	1	Weight reduction >20%; hemiparesis; cerebral hemorrhage	
**DMSO**	66	1	Weight reduction 5-10%; fur defects (decrease body care); reduced and delayed movement	
**DMSO**	76	1	Weight reduction 5-10%; critically moribund condition; extremely labored respiration; profound loss of vitality	
**DMSO**	77	1	Weight reduction >20%; seizures; automatisms; cerebral hemorrhage	
**DMSO**	78	1	Weight reduction 11-20%; fur defects (decrease body care); medium hydrocephalus; cerebral hemorrhage	
**DMSO**	83	1	Weight reduction >20%; seizures; automatisms	
**Lomitapide**	58	1	Weight reduction >20%; fur defects (decrease body care)	9
**Lomitapide**	66	1	Weight reduction 5-10%; critically moribund condition; extremely labored respiration; profound loss of vitality	
**Lomitapide**	69	1	Weight reduction >20%; seizures; automatisms	
**Lomitapide**	74	1	Weight reduction >20%; reduced and delayed movement	
**Lomitapide**	82	1	Weight reduction >20%; seizures	
**Lomitapide**	83	1	Weight reduction 5-10%; critically moribund condition; extremely labored respiration; profound loss of vitality	
**Lomitapide**	85	1	Weight reduction >20%; cerebral hemorrhage	
**Lomitapide**	148	1	Weight reduction 5-10%; fur defects (decrease body care); reduced and delayed movement; cerebral hemorrhage	
**Lomitapide**	185	1	Weight reduction 5-10%; critically moribund condition; extremely labored respiration; profound loss of vitality	

Supplementary Table 8. Genotype-based molecular milestones using LEGO model

	**Tumorigenic *in vivo***	**scRNA-Seq**	**DNA Methylome**	**Metabolome**	**Lipidome**	**Proteome**
**Shared Features**	Yes	Increased number of stem cells	Dynamic changes in methylation	Phospholipid; Glycolysis; Changes in metabolites regulating methylation (Serine, a-KG, 2-HG)	DG/TG increase; PC increase; Structural lipid decrease	EMT program; Lipid biosynthesis; increase in unfolded protein response
**PT**	Growth +; Infiltration +; Angiogenesis +	Astrocyte fate switch +	Methylation change +		DG/TG increase	AKT1 increase; mTOR increase
**PTCC**	Growth ++; Infiltration +; Angiogenesis +	Astrocyte fate switch ++; Activation of WNT pathway	Methylation change ++ MGMT methylated	Abnormal branched-chain amino acid metabolism	DG/TG increase O-PE decrease 1m	AKT1 mTOR CDK1/2/7 increase
**PTN**	Growth +; Infiltration +++; Angiogenesis +++	*HOX* gene activation; EMT increase Mesenchymal cell cluster	Methylation change +++; EMT and inflammation changes	Tryptophan low; Kynurenine high; Proline/hydroxyproline high	Increase of Ceramide; DG/TG increase	Collagen high; Mesenchymal signature; AKT1, mTOR and MAPK increase; IDH1 low; MGMT high

Abbreviations: α-KG, α-Ketoglutarate; 2-HG, 2-Hydoxyglutaric Acid; DG, Diacylglycerol; TG, Triacylglycerol; PC, Phosphatidylcholine; EMT, Epithelial-Mesenchymal Transition; O-PE, Ether Phosphatidylethanolamine; AKT1, AKT serine/threonine kinase 1; mTOR, Mammalian Target of Rapamycin; CDK, Cyclin Dependent Kinase; MAPK, Mitogen Activated Kinase-like Protein; IDH1, Isocitrate Dehydrogenase 1; MGMT, O6-Methylguanine-DNA Methyltransferase

Supplementary References

1 Uhlen, M. *et al.* Proteomics. Tissue-based map of the human proteome. *Science* **347**, 1260419 (2015). https://doi.org:10.1126/science.1260419

2 Varn, F. S. *et al.* Glioma progression is shaped by genetic evolution and microenvironment interactions. *Cell* **185**, 2184-2199 (2022). https://doi.org:10.1016/j.cell.2022.04.038

3 Johnson, K. C. *et al.* Single-cell multimodal glioma analyses identify epigenetic regulators of cellular plasticity and environmental stress response. *Nature Genetics* **53**, 1456-1468 (2021). https://doi.org:10.1038/s41588-021-00926-8

4 Sturm, D. *et al.* Hotspot Mutations in H3F3A and IDH1 Define Distinct Epigenetic and Biological Subgroups of Glioblastoma. *Cancer Cell* **22**, 425-437 (2012). https://doi.org:10.1016/j.ccr.2012.08.024

5 Wang, Q. *et al.* Tumor Evolution of Glioma-Intrinsic Gene Expression Subtypes Associates with Immunological Changes in the Microenvironment. *Cancer Cell* **32**, 42-56.e46 (2017). https://doi.org:10.1016/j.ccell.2017.06.003
