# Supplementary material for: A multidimensional atlas of human glioblastoma-like organoids reveals highly coordinated molecular networks and effective drugs
Source: NPJ Precis Oncol. 2024 Jan 26;8:19. doi: 10.1038/s41698-024-00500-5 (PMC10811239; doi:10.1038/s41698-024-00500-5)
Supplement: Supplementary file 2 — Reporting summary [file 41698_2024_500_MOESM2_ESM.pdf]

nature portfolio

Corresponding author(s): Haikun Liu

Last updated by author(s): Nov 30, 2023

# Reporting Summary

Nature Portfolio wishes to improve the reproducibility of the work that we publish. This form provides structure for consistency and transparency in reporting. For further information on Nature Portfolio policies, see our Editorial Policies and the Editorial Policy Checklist.

## Statistics

For all statistical analyses, confirm that the following items are present in the figure legend, table legend, main text, or Methods section.

| n/a | Confirmed | |
|---|---|---|
| ☐ | ☒ | The exact sample size (*n*) for each experimental group/condition, given as a discrete number and unit of measurement |
| ☐ | ☒ | A statement on whether measurements were taken from distinct samples or whether the same sample was measured repeatedly |
| ☐ | ☒ | The statistical test(s) used AND whether they are one- or two-sided<br>*Only common tests should be described solely by name; describe more complex techniques in the Methods section.* |
| ☒ | ☐ | A description of all covariates tested |
| ☐ | ☒ | A description of any assumptions or corrections, such as tests of normality and adjustment for multiple comparisons |
| ☐ | ☒ | A full description of the statistical parameters including central tendency (e.g. means) or other basic estimates (e.g. regression coefficient) AND variation (e.g. standard deviation) or associated estimates of uncertainty (e.g. confidence intervals) |
| ☐ | ☒ | For null hypothesis testing, the test statistic (e.g. *F*, *t*, *r*) with confidence intervals, effect sizes, degrees of freedom and *P* value noted<br>*Give P values as exact values whenever suitable.* |
| ☒ | ☐ | For Bayesian analysis, information on the choice of priors and Markov chain Monte Carlo settings |
| ☒ | ☐ | For hierarchical and complex designs, identification of the appropriate level for tests and full reporting of outcomes |
| ☒ | ☐ | Estimates of effect sizes (e.g. Cohen's *d*, Pearson's *r*), indicating how they were calculated |

*Our web collection on statistics for biologists contains articles on many of the points above.*

## Software and code

Policy information about availability of computer code

| | |
|---|---|
| Data collection | Living image (PerkinElmer) and QuickView Series Imaging system (Bio-Real) were used for bioluminescence imaging. ChemiDoc (Bio-Rad) for western blotting, TissueFAXS (TissueGnostics) and Zen (Zeiss) software were used for acquiring immunoflurescent and immunohistochemistry images. |
| Data analysis | ImageJ (1.53q, image processing), GraphPad Prism 9.0.0, GSEA (v4.2.3), Cytoscape (3.9.1), MaxQuant (v1.6.14.0), R (v4.1.2), Cell Ranger (v3.1.0), ElMaven (Elucidata), Limma (3.50.3, R package), missForest (1.5, R package), CHAMP (v2.24.0, R package), Fgsea (v1.20.0, R package), MetaboAnalystR (v3.2.0, R package), ChemRich(R package), VSN (v3.62.0, R package), Seurat (v3.1.5, R package), Velocyto (v0.17.17, R package), Scvelo (v0.2.2, R package), clusterProfiler (v4.2.2, R package). This study did not generate original code. |

For manuscripts utilizing custom algorithms or software that are central to the research but not yet described in published literature, software must be made available to editors and reviewers. We strongly encourage code deposition in a community repository (e.g. GitHub). See the Nature Portfolio guidelines for submitting code & software for further information.

## Data

Policy information about availability of data

All manuscripts must include a data availability statement. This statement should provide the following information, where applicable:

- Accession codes, unique identifiers, or web links for publicly available datasets
- A description of any restrictions on data availability
- For clinical datasets or third party data, please ensure that the statement adheres to our policy

ScRNA-seq, DNA methylation array and RNA-Seq data have been deposited at GEO under accession codes GSE213835, GSE213554 and GSE247890. Metabolomics and lipidomics data are available at the NIH Common Fund's National Metabolomics Data Repository (NMDR) website, the Metabolomics Workbench, (https://www.metabolomicsworkbench.org) where it has been assigned Study ID ST002284. The proteomics and phospho-proteomics data have been deposited to the ProteomeXchange Consortium via the iProX partner repository with the dataset identifier PXD036874. The DNA methylation data for human GBM can be accessed through GSE36278.

## Human research participants

Policy information about studies involving human research participants and Sex and Gender in Research.

| | |
|---|---|
| Reporting on sex and gender | *Use the terms sex (biological attribute) and gender (shaped by social and cultural circumstances) carefully in order to avoid confusing both terms. Indicate if findings apply to only one sex or gender; describe whether sex and gender were considered in study design whether sex and/or gender was determined based on self-reporting or assigned and methods used. Provide in the source data disaggregated sex and gender data where this information has been collected, and consent has been obtained for sharing of individual-level data; provide overall numbers in this Reporting Summary. Please state if this information has not been collected. Report sex- and gender-based analyses where performed, justify reasons for lack of sex- and gender-based analysis.* |
| Population characteristics | *Describe the covariate-relevant population characteristics of the human research participants (e.g. age, genotypic information, past and current diagnosis and treatment categories). If you filled out the behavioural & social sciences study design questions and have nothing to add here, write "See above."* |
| Recruitment | *Describe how participants were recruited. Outline any potential self-selection bias or other biases that may be present and how these are likely to impact results.* |
| Ethics oversight | *Identify the organization(s) that approved the study protocol.* |

Note that full information on the approval of the study protocol must also be provided in the manuscript.

# Field-specific reporting

Please select the one below that is the best fit for your research. If you are not sure, read the appropriate sections before making your selection.

☒ Life sciences ☐ Behavioural & social sciences ☐ Ecological, evolutionary & environmental sciences

For a reference copy of the document with all sections, see nature.com/documents/nr-reporting-summary-flat.pdf

# Life sciences study design

All studies must disclose on these points even when the disclosure is negative.

| | |
|---|---|
| Sample size | Sample sizes were estimated empirically, and were higher than the lowest number for statistical analysis. |
| Data exclusions | Two four-month-old metabolic profiling samples (one PT and one PTN) were excluded from downstream analysis since the signals of internal standards were much lower and blank controls, which we think could due to LC/MS measurement error. One WT RNA sequencing sample failed sequencing and was excluded from the following analysis. |
| Replication | Data shown from representative experiments were repeated with similar results in at least 3 independent experiments, unless otherwise indicated by sample size. All attempts at replication were successful. |
| Randomization | Mice were randomly assigned to different groups and organoids were randomly selected for specific assays and drug screen. |
| Blinding | Blinding was not relevant for the experiments, because the readouts for the measurements were not determined by human judgment. |

# Reporting for specific materials, systems and methods

We require information from authors about some types of materials, experimental systems and methods used in many studies. Here, indicate whether each material, system or method listed is relevant to your study. If you are not sure if a list item applies to your research, read the appropriate section before selecting a response.

## Materials & experimental systems

| n/a | Involved in the study |
|---|---|
| ☐ | ☒ Antibodies |
| ☐ | ☒ Eukaryotic cell lines |
| ☒ | ☐ Palaeontology and archaeology |
| ☐ | ☒ Animals and other organisms |
| ☒ | ☐ Clinical data |
| ☒ | ☐ Dual use research of concern |

## Methods

| n/a | Involved in the study |
|---|---|
| ☒ | ☐ ChIP-seq |
| ☒ | ☐ Flow cytometry |
| ☒ | ☐ MRI-based neuroimaging |

## Antibodies

Antibodies used

Rabbit monoclonal anti PTEN, Cell Signaling Technology 9559
Mouse monoclonal anti P53, Thermo Fisher Scientific MA512557
Mouse monoclonal anti P15/P16, Santa Cruz, sc-377412
Mouse monoclonal anti NF1 (Reuss et al. 2014), produced by the Antibody core facility of DKFZ NF1-146/29/25
Rabbit monoclonal anti GAPDH, Cell Signaling Technology 2118
Rabbit monoclonal anti β-Tubulin, Cell Signaling Technology 2128
Goat anti-Rabbit HRP, Cell Signaling Technology 7074
Horse anti-Mouse HRP, Cell Signaling Technology 7076
Rabbit monoclonal anti Ki67, Cell Signaling Technology 9129
Chicken polyclonal anti GFP, Abcam ab13970
Rabbit monoclonal anti CD31, Abcam ab182981
Mouse monoclonal anti GFAP, Cell Signaling Technology 3670
Mouse monoclonal anti Nestin, Cell Signaling Technology 33475
Rabbit polyclonal anti SOX2, Abcam ab97959
Mouse monoclonal anti Tubulin B3 (TUJ1), Biolegend 801202
Mouse anti BrdU, BD 347580
Goat anti-Chicken Alexa Fluor 488, Thermo Fisher Scientific A11039
Donkey anti-Rabbit Alexa Fluor 555, Thermo Fisher Scientific A31572
Donkey anti-Mouse CF® 633, Sigma-Aldrich SAB4600128
Goat anti-Rabbit Alexa Fluor 555, Thermo Fisher Scientific A21428
Goat anti-Rabbit Alexa Fluor 647, Thermo Fisher Scientific A21235
Rabbit polyclonal anti FABP7 (BLBP), Abcam ab32423
Rabbit monoclonal anti SOX9, Abcam ab185966
Mouse monoclonal anti Doublecortin (DCX), Santa cruz, sc-291390

Validation

For Mouse monoclonal anti NF1, details regarding the manufacturering process can be found in Reuss et al. Acta Neuropathol 2014.
For other antibodies, validation and references are provided on manufacturer's website.
Rabbit monoclonal anti PTEN, Cell Signaling Technology 9559, 673 studies;
Mouse monoclonal anti P53, Thermo Fisher Scientific MA512557, 159 studies;
Mouse monoclonal anti P15/P16, Santa Cruz, sc-377412, 40 studies;
Rabbit monoclonal anti GAPDH, Cell Signaling Technology 2118, 6148 studies;
Rabbit monoclonal anti β-Tubulin, Cell Signaling Technology 2128, 679 studies;
Goat anti-Rabbit HRP, Cell Signaling Technology 7074, 11557 studies;
Horse anti-Mouse HRP, Cell Signaling Technology 7076, 6684 studies;
Rabbit monoclonal anti Ki67, Cell Signaling Technology 9129, 257 studies;
Chicken polyclonal anti GFP, Abcam ab13970, 2684 studies;
Rabbit monoclonal anti CD31, Abcam ab182981, 63 studies;
Mouse monoclonal anti GFAP, Cell Signaling Technology 3670, 548 studies;
Mouse monoclonal anti Nestin, Cell Signaling Technology 33475, 22 studies;
Rabbit polyclonal anti SOX2, Abcam ab97959, 529 studies;
Mouse anti BrdU, BD 347580, 1030 studies;
Mouse monoclonal anti Tubulin B3 (TUJ1), Biolegend 801202, 543 studies;
Goat anti-Chicken Alexa Fluor 488, Thermo Fisher Scientific A11039, 1859 studies;
Donkey anti-Rabbit Alexa Fluor 555, Thermo Fisher Scientific A31572, 1555 studies;
Donkey anti-Mouse CF® 633, Sigma-Aldrich SAB4600128, 4 studies;
Goat anti-Rabbit Alexa Fluor 555, Thermo Fisher Scientific A21428, 1425 studies;
Goat anti-Rabbit Alexa Fluor 647, Thermo Fisher Scientific A21235, 1452 studies;
Rabbit polyclonal anti FABP7 (BLBP), Abcam ab32423, 89 studies;
Rabbit monoclonal anti SOX9, Abcam ab185966, 179 studies;
Mouse monoclonal anti Doublecortin (DCX), Santa cruz, sc-291390, 119 studies.

## Eukaryotic cell lines

Policy information about cell lines and Sex and Gender in Research

| | |
|---|---|
| Cell line source(s) | GFP+ WT iPSC is purchased from Coriell Institute (Cat#AICS-0036-006), another human iPSC line was provided by RUCDR Infinite Biologics (RUID: 06C53141), mutant iPSCs were generated in this study. The Pten/Trp53 KO mouse glioma stem cells (BTSC, mGB2) were described previously (doi: 10.3390/cancers13020230). |
| Authentication | None of the cell lines were authenticated. |
| Mycoplasma contamination | Cell lines were tested negative for mycoplasma contamination. |
| Commonly misidentified lines (See ICLAC register) | *Name any commonly misidentified cell lines used in the study and provide a rationale for their use.* |

## Animals and other research organisms

Policy information about studies involving animals; ARRIVE guidelines recommended for reporting animal research, and Sex and Gender in Research

| | |
|---|---|
| Laboratory animals | four- to six-week-old NOD/SCID and C57/BL6N mice |
| Wild animals | No wild animals were used in this study |
| Reporting on sex | female |
| Field-collected samples | No field samples were collected in this study |
| Ethics oversight | All mouse experiments were conducted under Shanghai Institutional Animal Care and Use Committee (IACUC) guidelines and an approved IACUC protocol of ShanghaiTech University (#20201208001). |

Note that full information on the approval of the study protocol must also be provided in the manuscript.
